# Phosphoproteome reveals molecular mechanisms of aberrant rhythm in neurotransmitter‐mediated islet hormone secretion in diabetic mice

**DOI:** 10.1002/ctm2.890

**Published:** 2022-06-27

**Authors:** Yunqiang He, Qi Fu, Min Sun, Yu Qian, Yucheng Liang, Jie Zhang, Rui Gao, Hemin Jiang, Hao Dai, Yuwei Liu, Xinyu Xu, Heng Chen, Kuanfeng Xu, Tao Yang

**Affiliations:** ^1^ Department of Endocrinology and Metabolism The First Affiliated Hospital of Nanjing Medical University Nanjing China; ^2^ Oxford Centre for Diabetes Endocrinology and Metabolism, Radcliffe Department of Medicine, University of Oxford Oxford UK

**Keywords:** diabetes mellitus, islet dysfunction, neurotransmitter, parasympathetic nerve, phosphoproteomics, sympathetic nerve

## Abstract

**Background:**

Acetylcholine (ACh) and norepinephrine (NE) are representative neurotransmitters of parasympathetic and sympathetic nerves, respectively, that antagonize each other to coregulate internal body functions. This also includes the control of different kinds of hormone secretion from pancreatic islets. However, the molecular mechanisms have not been fully elucidated, and whether innervation in islets is abnormal in diabetes mellitus also remains unclear.

**Methods and results:**

Immunofluorescence colocalization and islet perfusion were performed and the results demonstrated that ACh/NE and their receptors were highly expressed in islet and rapidly regulated different hormones secretion. Phosphorylation is considered an important posttranslational modification in islet innervation and it was identified by quantitative proteomic and phosphoproteomic analyses in this study. The phosphorylated islet proteins were found involved in many biological and pathological processes, such as synaptic signalling transduction, calcium channel opening and insulin signalling pathway. Then, the kinases were predicted by motif analysis and further screened and verified by kinase‐specific siRNAs in different islet cell lines (αTC1‐6, Min6 and TGP52). After functional verification, Ksr2 and Pkacb were considered the key kinases of ACh and NE in insulin secretion, and Cadps, Mlxipl and Pdcd4 were the substrates of these kinases measured by immunofluorescence co‐staining. Then, the decreased expression of receptors, kinases and substrates of ACh and NE were found in diabetic mice and the aberrant rhythm in insulin secretion could be improved by combined interventions on key receptors (M3 (pilocarpine) or α2a (guanfacine)) and kinases (Ksr2 or Pkacb).

**Conclusions:**

Abnormal innervation was closely associated with the degree of islet dysfunction in diabetic mice and the aberrant rhythm in insulin secretion could be ameliorated significantly after intervention with key receptors and kinases in the early stage of diabetes mellitus, which may provide a promising therapeutic strategy for diabetes mellitus in the future.

## Graphical abstract

1. Rapid regulation of neurotransmitters in islet is exerted by altering proteins phosphorylation rather than expression levels.
2. Proteins phosphorylation modulated by kinases play an important role in neurotransmitters ACh and NE mediated islet hormones secretion.
3. Impaired innervation and aberrant rhythm of insulin secretion were found in islet of diabetic mice, which could be ameliorated after intervention with key receptors and kinases.

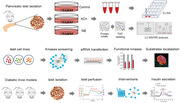

## 1 INTRODUCTION

The pancreatic islet is composed of various endocrine cells that play critical roles in controlling blood glucose homeostasis and energy metabolism by secreting different islet hormones.^1^ It has been reported that the islet is innervated by abundant neural fibres scattered in the pancreas that form a complex neural network to regulate hormone secretion through synapses and neurotransmitters under various physiological conditions.^2^, ^3^ Neural signals represent a natural route from the brain to target islets to influence islet function by regulating the secretion of various endocrine hormones apart from the conventional route of circulation.^4^ Normal innervation in the pancreatic islet is closely associated with the maintenance of islet function.^4^, ^5^ Although rich islet innervation has been described as far back as the discovery of islets by Paul Langerhans, the specific molecules and mechanisms in islet innervation patterns also remain unclear.

Nerves transmit brain signals through synapses and neurotransmitters to achieve rapid regulation of the secretion of various islet hormones in response to physiological cues.^6^, ^7^ The autonomic nervous system provides among the most important regulatory nerves in the islet, mainly consisting of sympathetic and parasympathetic nerves.^8^ These two types of nerves play important roles in regulating the secretion of different islet hormones through various neurotransmitters, such as norepinephrine (NE) and acetylcholine (ACh).^9^, ^10^, ^11^ The neurotransmitters NE and ACh are also the main transmitters of two efferent nerves (noradrenergic nerve and cholinergic nerve) in the brain.^12^ These two antagonistic neurotransmitters released by synapses in islets have advantages in rapidly regulating the secretion of various islet hormones under different conditions.^13^ Additionally, previous evidence has suggested that both NE receptors and muscarinic receptors of ACh are G protein‐coupled receptors (GPCRs) and that neurotransmitters in the pancreatic islet mainly act as ligands to bind with various receptor subtypes to achieve different physiological effects.^14^, ^15^, ^16^ However, the distribution of various receptor subtypes of neurotransmitter ACh and NE in different islet endocrine cells is unknown, and the molecular mechanisms underlying rapid neurotransmitter‐mediated hormone secretion are also unidentified.

Phosphorylation as the most common posttranslational modification is also an important downstream pathway of GPCRs in intracellular nerve signal transduction.^17^, ^18^ The reversible processes of phosphorylation and dephosphorylation are closely related to the activation of islet proteins and play critical roles in controlling neurotransmitter‐mediated islet hormone secretion.^19^, ^20^ Aberrant phosphorylation of islet proteins might participate in the initiation and development of diabetes mellitus.^21^ Furthermore, emerging evidence has also demonstrated that abnormal innervation and unbalanced activity of the autonomic nervous system are found in the islets of patients with a variety of metabolic syndromes.^22^, ^23^ Impaired glucose tolerance and blood glucose homeostasis imbalance were manifested in cholinergic and adrenergic receptor‐specific knockout mice.^24^, ^25^ Clinical studies have also revealed that decreased expression of the cholinergic receptor M3 caused by mutations in the single nucleotide polymorphisms locus of the coding gene *CHRM3* could be considered a high‐risk factor for diabetes mellitus.^26^ Thus, the investigation of specific molecular mechanisms in neuromodulation abnormalities and the intervention of key targets in islets are important issues for current diabetes research.

Islet cell dysfunction patterns, such as abnormal insulin secretory rates or pulsatile secretion loss to neurotransmitter stimuli, are closely related to aberrant neural signal transduction of the receptors in islets.^27^ However, most of the current studies have only focused on the intervention of various neurotransmitter receptors, and there have been few studies observing the downstream signalling pathways of different receptor subtypes.^28^ Therefore, in this study, mass spectrometry (MS)‐based quantitative proteomic and phosphoproteomic analyses were both performed to reveal the molecular mechanisms of neurotransmitter‐mediated islet hormone secretion in mouse islets. Various receptor subtypes, kinases and substrate proteins of the antagonistic neurotransmitters ACh and NE were further investigated in different islet endocrine cells and diabetic mice with diverse islet functions. Furthermore, aberrant insulin secretion rhythm could be improved after combined intervention with key receptors and kinases, which could provide a potential therapeutic method for diabetes mellitus in the future.

## 2 MATERIALS AND METHODS

### 2.1 Animals

Male ICR mice and C57BL/6 mice (8 weeks) were purchased from GemPharmatech Biotechnology (Nanjing, Jiangsu). Female NOD mice, male ob/ob mice and db/db mice were obtained from the Model Animal Research Center of Nanjing University. The high‐fat diet (HFD) mice models were established in ICR mice and C57BL/6 mice by feeding with a high‐fat diet (D12492, Research Diets, 60% fat) for 4 weeks. All rats were fed ad libitum with a regular 12‐h light/dark cycle. The protocols of animal experiments in this study were approved by the Animal Care and Use Committee of Nanjing Medical University.

### 2.2 Reagents and antibodies

β‐mercaptoethanol, aprotinin, ACh, Histopaque‐1077 and 1119 were obtained from Sigma‐Aldrich. Collagenase V was provided by Roche Diagnostics (Indianapolis, IN, USA). L‐Norepinephrine was purchased from J&K Scientific (Beijing, China). Lipofectamine 3000 was obtained from Invitrogen (California, USA). The antibodies to TH, NET and c‐Fos were from Cell Signaling Technology (Boston, USA). The antibodies to Mlxipl and Pdcd4 were purchased from Proteintech (Chicago, IL, USA). The antibodies to Synapsin, AChE, VAChT, Cadps, Sik2, TH (phospho S31), muscarinic receptors (M1‐M5) and adrenergic receptors (α1a‐α2c) were provided by Abcam (Cambridge, UK). Anti‐Cadps (phospho S259) monoclonal antibody was synthesized and produced by SGE Biotech (Suzhou, China). The phosphorylated antibodies including Sik2 (Phos S358), Mlxipl (Phos S196) and Pdcd4 (Phos S313) were synthesized by PTM BioLab (Hangzhou, China). The information on antibodies used in this study is listed in Table 1.

**TABLE 1:** The information on antibodies

Antibodies	Source	Identifier
Synapsin	Abcam	ab64581
Tyrosine Hydroxylase	Cell Signaling Technology	58844S
Noradrenaline transporter	Abcam	ab211463
Vesicular Acetylcholine Transporter	Abcam	ab235201
Acetylcholinesterase	Abcam	ab183591
Insulin	Abcam	ab6995
Glucagon	Abcam	ab92517
Somatostatin	Abcam	ab108456
CD31 (PECAM‐1)	Cell Signaling Technology	3528S
Muscarinic acetylcholine Receptor M1	Abcam	ab180636
Muscarinic acetylcholine Receptor M2	Abcam	ab109226
Muscarinic acetylcholine Receptor M3	Abcam	Ab87199
Muscarinic acetylcholine Receptor M4	Abcam	ab77956
Muscarinic acetylcholine Receptor M5	Abcam	ab186830
Anti‐αlpha 1a adrenergic receptor	Abcam	ab137123
Anti‐αlpha 1b adrenergic receptor	Abcam	ab169523
Anti‐αlpha 1d adrenergic receptor	Abcam	ab84402
Anti‐αlpha 2a adrenergic receptor	Abcam	ab85570
Anti‐αlpha 2b adrenergic receptor	Santa Cruz Biotechnology	sc‐65211
Anti‐αlpha 2c adrenergic receptor	Abcam	ab167433
Anti‐Cadps	Abcam	ab32011
Anti‐c‐Fos	Abcam	ab208942
Anti‐Mlxipl	Proteintech	13256‐1‐AP
Anti‐Pdcd4	Proteintech	12587‐1‐AP
Anti‐Sik2	Abcam	ab53423
Phos‐TH (S31)	Abcam	ab254031
Phos‐c‐Fos (S362)	Affinity	AF3053
Phos‐Cadps (S259)	SGE Biotech	SG820001
Phos‐Sik2 (S358)	PTM BioLab	CTM‐233
Phos‐Mlxipl (S196)	PTM BioLab	CTM‐236
Phos‐Pdcd4 (S313)	PTM BioLab	CTM‐231
Goat anti‐Mouse IgG (H+L) Antibody Alexa Fluor 488	Cell Signaling Technology	4408S
Goat anti‐Mouse IgG (H+L) Antibody Alexa Fluor 594	Abcam	ab150116
Goat anti‐Rabbit IgG (H+L) Antibody Alexa Fluor 488	Cell Signaling Technology	4412S
Goat anti‐Rabbit IgG (H+L) Antibody Alexa Fluor 594	Abcam	ab150080
Goat anti‐Rat IgG (H+L) Antibody Alexa Fluor 488	Cell Signaling Technology	4416S
Goat anti‐Rat IgG (H+L) Antibody Alexa Fluor 594	Abcam	ab150160
Anti‐β‐actin	Cell Signaling Technology	3700S

### 2.3 Islet isolation and transmitters stimulation

Mouse pancreatic islets were isolated according to the protocol described previously.^29^ The pancreas was inflated fully with collagenase V through the common bile duct and separated for digestion at 37°C. After digestion, the pancreatic tissues were washed with Hank's solution and purified with density gradient centrifugation (Histopaque 1119 and 1077). Isolated islets were handpicked and cultured in RPMI‐1640 (10% fetal bovine serum). Then, antagonistic neurotransmitters ACh and NE were used to treat the islets after starvation in vitro. The concentration of insulin, glucagon and somatostatin in the supernatant was measured by ELISA.

### 2.4 Islet perfusion and islet hormones measurements

Islet perfusion was performed to reveal the rapid regulatory effects of these two antagonistic neurotransmitters on the secretion of different islet hormones in this study. The islet perfusion apparatus included a single channel heater control (37°C), a peristaltic pump, a perfusion chamber and an automatic sample collector. A total of 100 isolated mouse islets were loaded on the filter membrane in the perfusion chamber using a P200 pipette. And the liquid flow rate of perfusion was maintained at 300–400 μl/min by adjusting the amount of P4 beads in the chamber. The islets were stimulated with KRBH buffer (5.5 mM glucose) containing neurotransmitters ACh and NE, and the perfusate was also collected to measure the secretion levels of different islet hormones. The concentration of insulin (Min6), glucagon (α TC1‐6) and somatostatin (TGP52) were also measured by ELISA.

### 2.5 Proteome and phosphoproteome analyses

#### 2.5.1 Sample preparation

The proteome and phosphoproteome analyses were performed in APTBIO (Shanghai, China). Isolated murine islets were treated with neurotransmitters NE or ACh for 30mins in vitro and lysed in SDT buffer (4% SDS, 100 mM Tris‐HCl and 1Mm DTT, pH 7.6, 30 μl buffer per 100 islets). The fully lysed protein samples were boiled at 100°C for 15 min and centrifuged at 14 000 × g for 40 min. The supernatant was filtered using a 0.22‐μm filter and stored at ‐80°C. The protein concentration was determined by a BCA protein assay kit (Thermo Fisher Scientific, MA, USA).

#### 2.5.2 Protein digestion and TMT labelling

Protein digestion was performed according to the protocol reported previously. 30 μl SDT buffer was used per 200 μg of protein. UA buffer (8 M Urea and 150 mM Tris‐HCl) was used to remove the DTT and detergent at 14 000 × g for 10 min. After washing with UA buffer and DS buffer, the proteins were digested with trypsin at 37°C overnight. Then, the peptides were collected and desalted by C18 Cartridges and concentrated by vacuum centrifugation. The peptides were dissolved with 0.1% formic acid and measured by using an ultraviolet spectrophotometer at 280 nm. Finally, the peptides were labelled by tandem mass tag (TMT) Mass Tag Labeling Kits according to the manufacturer's instructions (Thermo Fisher Scientific).

#### 2.5.3 Phosphorylated peptides enrichment

The labelled peptides were concentrated by the vacuum concentrator and then resuspended with a 500 μl loading buffer. Then, TiO_2_ beads were added to the solution and stirred well for 40 min. The beads were collected after centrifugation at 5000 × g for 1 min at room temperature. After centrifuging with beads with washing buffer Ⅰ (3% TFA, 30% ACN,) and Ⅱ (80% CAN, 0.3 TFA). The elution buffer was added to elute and collect the phosphopeptides for liquid chromatography‐tandem mass spectrometry (LC‐MS/MS) analysis.

#### 2.5.4 LC‐MS/MS analysis

Enriched phosphorylated peptides were separated using high‐performance liquid chromatography Easy nLC1000 (HPLC; Thermo Fisher Scientific). The mixed peptides were loaded on the reverse phase trap column (Acclaim PepMap100, nanoViper C18) that connected to the reversed‐phase analytical column in buffer with 0.1% formic acid and separated in buffer was 0.1% formic acid. Then, the separated peptide fractions were analyzed by LC‐MS/MS based on a Q Exactive mass spectrometer coupled to Easy nLC. MS data were acquired as previously described by using a data‐dependent top 10 method dynamically and choosing abundant precursor ions from the survey scan (300–1800 *m/z*) for higher collision dissociation fragmentation.

#### 2.5.5 Database search and data analysis

Raw MS/MS data were analyzed for database search using the Andromeda engine in the MaxQuant environment (version 1.6.2.0) according to the protocol described previously. The Mus Murine UniProt FASTA database was used to match with the MS/MS spectra in this study with an FDR < 1% at the level of peptides, proteins, and modifications. The TMT labelled proteome analysis was also performed in the MaxQuant environment. The newly generated MS proteomics data have been deposited in the ProteomeXchange database via the PRIDE partner repository with the following accession number (PXD031491). All other data in this study supporting the findings are available within the article and supplementary files.

#### 2.5.6 Bioinformatics data analysis

The Perseus software was used for bioinformatics data analysis of proteome and phosphoproteome in this study. Statistical analysis was performed on logarithmized intensities for the values found to be quantified. Student's t‐test was used to identify significantly phosphorylate proteins and phosphopeptides between two different groups. MetaboAnalyst 3.0 and the Java Treeview software were used for hierarchical clustering analysis. Categorical annotation was added in the form of gene ontology (GO) biological process, cellular component, molecular function, Kyoto Encyclopedia of Genes and Genomes (KEGG) pathways and kinase substrate motifs in the Perseus environment. The analyses of GO enrichment and KEGG pathway enrichment were performed according to Fisher's exact test. Enrichment analysis was performed by using a group‐based phosphorylation score algorithm (GPS 2.0) to identify statistically significant enriched kinase‐substrates motifs and predict kinase‐specific phosphorylation sites. The corresponding score and *p*‐value were assigned for each kinase‐substrate motif.

#### 2.5.7 Motif analysis

Protein phosphorylation was mainly based on the recognition of the substrate‐specific conservation motifs of amino acids by protein kinases (PKs). Motif analysis in this study was performed by using Motif‐X algorithm analysis software (http://motif‐x.med.harvard.edu/) to identify the kinase‐specific phosphosites and the relationship between kinases and substrates. The screening parameters were set to the frequency of a certain phosphosite > 50% (*p*‐value < 10^−5^) identified by mass spectrometry in sequence windows. The upstream kinases of different phosphosite predicted by mass spectrometry were analyzed by GPS 2.0 software and further screened through a protein interaction database to improve the prediction accuracy.^30^ Serine, threonine and tyrosine were selected as the central amino acids and the generated motifs width was set as seven amino acids. Gene set enrichment analysis was also used to analyze the activities of predicted upstream kinases to evaluate the regulation of kinases on different substrates accurately. All the kinases were further verified in mouse islets and different pancreatic cell lines in this study.

#### 2.5.8 Cell culture and transfection

Three kinds of pancreatic cell lines were used to investigate the effects of kinases on islet hormones (insulin, glucagon and somatostatin) secretion after neurotransmitters administration. Min6 and α TC1‐6 cell lines were kindly provided by Professor Zhuoxian Meng from Zhejiang University and Professor Zheng Chen from Harbin Institute of Technology, respectively. TGP52 cell line was purchased from American Type Culture Collection (ATCC, Bethesda, MD). All these cell lines were cultured in the mediums supplemented with 10% FBS in 5% CO_2_ at 37°C. The cell lines were transfected with Lipofectamine 3000 and interfered with siRNAs (small interfering RNA, sequences shown in Figure S5C) according to the manufacturer's instructions to determine the key kinases involved in the regulation of neurotransmitters ACh and NE on islet hormones secretion.

#### 2.5.9 Immunohistochemical and immunofluorescence staining

Immunohistochemical and immunofluorescence staining were performed according to the manufacturer's instructions and protocols described previously. The diaminobenzidine was used to reveal the conjugates of primary and secondary antibodies. The immunofluorescence co‐staining was also performed with different antibodies incubated together in the section. And, the secondary antibodies from different species were used to combine with the primary antibodies separately. At least six different regions were obtained from a separate section in each group and analyzed on a double‐blinded method.

#### 2.5.10 Quantitative real‐time polymerase chain reaction

Total RNA was extracted from islets or cells with TRIzol reagent according to the manufacturer's instructions. cDNA was prepared by using the reverse transcription system kit (Takara PrimeScript RT Master Mix, Clontech Laboratories, USA). Quantitative real‐time polymerase chain reaction (RT‐PCR) was performed by using SYBR Premix Ex Taq Ⅱ Kit (Clontech Laboratories, USA) through Step One Plus RT‐PCR System (Applied Biosystems, USA) to detect the relative mRNA levels of genes. β‐actin was used as the internal control and mRNA expression levels were measured by using the comparative ΔCt method. All the sequences of primers in this study are listed in Table 2 as follows.

**TABLE 2:** The primer sequences of different receptor subtypes of norepinephrine (NE) and acetylcholine (ACh)

Gene	Forward primer (5′→3′)	Reverse primer (5′→3′)
Receptors (ACh)		
Chrm1	GAACTCCCAGAAACTGTTGGA	TTGATGCAACTGATCCGACT
Chrm2	GGCCTCCAACATGAGAGATG	TCACCATTCTGACCTGACGA
Chrm3	GACCATCATCGGCAACATC	AGAGCCCAGCGGTTCATAAT
Chrm4	GCCATGCTAAGCCGAGATAC	ACCATCACCTTTCCCTCCTT
Chrm5	TTGGCTTGCACTCGACTATG	GGATCTGGCACTCATCAGGT
Receptors (NE)		
Adra1a	GCCAGGATACGTGCTGTTCT	GTGGATACGGAGCGTCACTT
Adra1b	CGCCCACCAACTACTTCATT	ATGGCACATAGGCTCAGGAT
Adra1d	TGGGCTATGCGATCTTCTCT	GGATTTCCTTTGGCGCTAGT
Adra2a	CATCAGCCTTGACCGCTAC	TCTGGTCGTTGATCTTGCAG
Adra2b	TGAGCAAGAATGTGGGTGTG	TTGCAGTAGCCAATCCAGAA
Adra2c	GAGCTTGGGAGCTTGATCTG	CTTGTAGCAGCATGGCAGAG
β‐actin	GTGGGCCGCTCTAGGCACCAA	CTCTTTGATGTCACGCACGATTTC

#### 2.5.11 Western blot analysis

Protein was extracted from the mouse pancreatic islet by using RIPA lysis buffer and measured by a BCA assay kit (Beyotime, Jiangsu, China). A total of 30 μg protein was separated by 10% sodium dodecyl sulfate‐polyacrylamide gel electrophoresis and transferred onto polyvinylidene fluoride membranes. Then, the membranes were blocked with nonfat milk and incubated with primary antibodies at 4°C overnight. HRP‐conjugated IgG secondary antibodies were used to combine with the primary antibodies and visualized by an ECL imaging system (Bio‐Rad, California, USA).

### 2.6 Statistical analysis

The quantitative analysis of staining results in this study was calculated by fluorescence intensity and optical density measurement with Image J (National Institutes of Health, USA). All the experiments in this study were performed in at least biological triplicates. The data were analyzed by using SPSS 20.0 (Chicago, IL, USA) and presented as the mean ± standard deviation. The correlation was also quantified by Pearson's r for the parametric data analysis. Statistical analysis between groups was performed by using Student's unpaired t‐test or one‐way analysis of variance and *p* < 0.05 was considered statistically significant.

## 3 RESULTS

### 3.1 Antagonistic neurotransmitters ACh and NE in different islet endocrine cells elicit diverse responses

**FIGURE 1:**
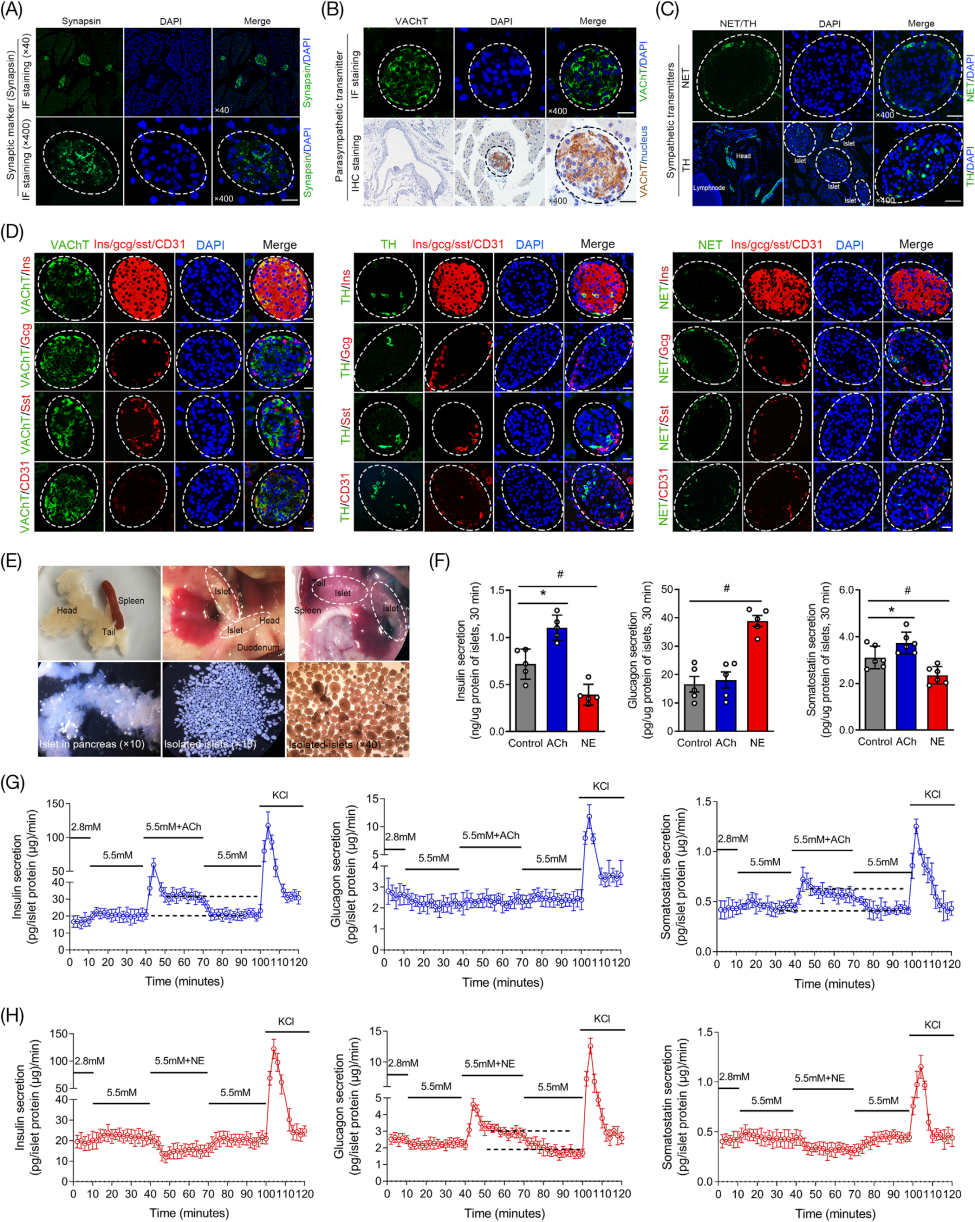
The distributions of the neurotransmitters acetylcholine (ACh) and norepinephrine (NE) in pancreatic islets and their regulatory effects on the secretion of different islet hormones. (A) The distribution of synapses in the pancreas and islets was revealed by immunofluorescence staining of the specific synaptic protein (synapsin, green). (B,C) The expression and distribution of specific markers of ACh (VAChT) and NE (NET and TH) in the pancreas and islets. (D) The distributions of specific markers of ACh and NE were demonstrated by immunofluorescence multistaining and colocalization analysis with pancreatic α‐cells, β‐cells, δ‐cells and vascular endothelial cells in islets. x400 magnification, scale bar: 25 μm. (E) Pancreatic islets distributed in the head and tail of the pancreas under an optical microscope and highly purified mouse islets were isolated and treated with ACh and NE for a short time in vitro. (F) The secretion levels of different islet hormones (insulin, glucagon and somatostatin) were measured in different groups. (G,H) The instantaneous secretion rates of different islet hormones were also revealed by islet perfusion under stimulation with the neurotransmitters ACh and NE. *p < 0.05: ACh group versus the control group, #*p* < 0.05: NE group versus the control group

The autonomic nerves in pancreatic islets mainly modulate islet hormone secretion through synapses and neurotransmitters. Immunofluorescence staining was first performed to reveal the synapses of autonomic nerves in the pancreas, and the results showed that synapsin (a synaptic marker) was highly expressed in the islets (Figure 1A). Then, immunofluorescence staining and immunohistochemical staining of specific markers of ACh (VAChT/AChE) and NE (NET/TH) were both performed, and the results demonstrated that the neurotransmitters ACh and NE were mainly distributed in the islets (Figure 1B,C and Figure S1A), while AChE was not activated in the normal mice (Figure S1B,C). Additionally, immunofluorescence co‐staining showed that VAChT, TH and NETs were mainly located in β‐cell, while they were not obvious in α‐cell, δ‐cell or vascular endothelial cell (Figure 1D). As shown in Figure 1E,F, isolated murine islets were stimulated with ACh and NE in vitro, and the results revealed that ACh could significantly increase insulin and somatostatin secretion but almost failed to affect the secretion of glucagon (α‐cell). The sympathetic neurotransmitter NE had a significant effect on glucagon secretion but inhibited hormone secretion by β‐cell and δ‐cell. In addition, to eliminate the mutual interference between various islet hormones, islet perfusion was also performed to accurately evaluate the rapid regulation of neurotransmitters on different islet endocrine cells (Figure 1G,H). The results were consistent with the previous culture experiment in vitro in this study. Neurotransmitter ACh can rapidly promote insulin and somatostatin secretion, and the regulatory effect was more significant in β cells. Additionally, the secretion level of glucagon was rapidly elevated after NE stimulation. We also found that there was a significant “platform phase” in islet hormone secretion that was modulated by the neurotransmitters ACh and NE.

### 3.2 Receptor subtypes of ACh and NE expressed in different islet endocrine cells

**FIGURE 2:**
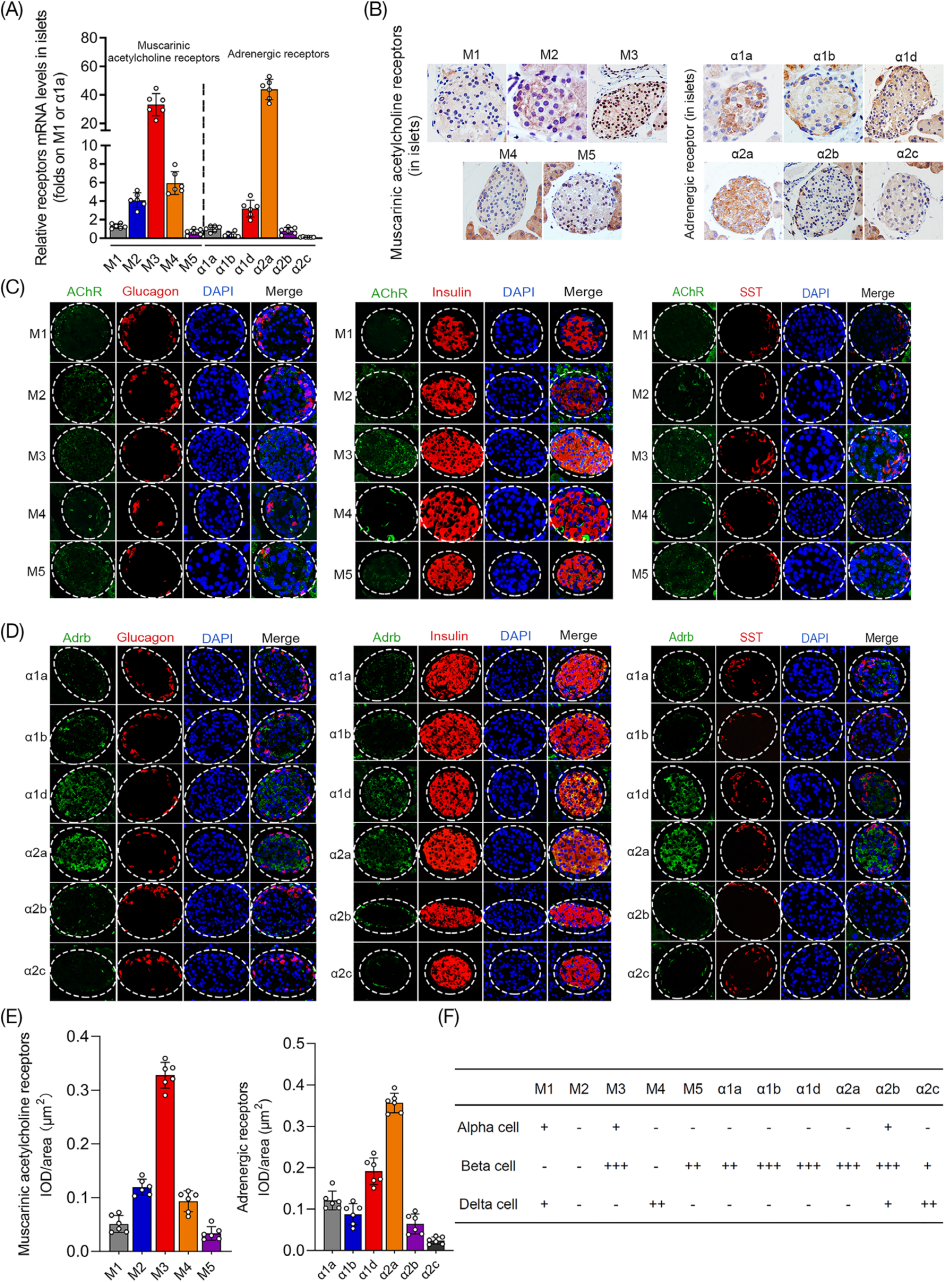
Quantitative analyses of various receptor subtypes of acetylcholine (ACh) (muscarinic receptors) and norepinephrine (NE) (adrenergic receptors) expressed in murine islets. (A) The expression levels of different receptor subtypes of ACh and NE were measured in murine islets by real‐time polymerase chain reaction (RT‐PCR) analysis. (B) Immunohistochemical staining of five kinds of muscarinic receptor subtypes of ACh and six kinds of adrenergic receptors of NE in pancreatic islet. (C,D) The expression and distribution of various ACh and NE receptors in islets were revealed by immunofluorescence multistaining and colocalization analysis with different endocrine cells (alpha‐cell, beta‐cell and delta‐cell). (E) The expression levels of different receptors of ACh and NE in pancreatic islets were measured by the mean IOD/area (μm^2^). (F) Quantitative analysis of various receptors of ACh and NE expressed in alpha‐cell, beta‐cell and delta‐cell. “ + ”: costained area accounts for positive area < 25%; “ ++ ”: 25% < costained area accounts for positive area < 50%; “ +++ ”: costained area accounts for positive area ≥ 50%

Neurotransmitters in the pancreatic islet mainly act as ligands and bind with different receptor subtypes to achieve various physiological effects. In this study, the expression and distribution of different receptors of the neurotransmitters ACh and NE were evaluated by real‐time RT‐PCR, immunohistochemical staining and immunofluorescence co‐staining. The mean integrated optical density (IOD)/area was also used to quantify the expression of different receptors. As shown in Figure 2A,B, the results showed that the receptors of ACh and NE were widely expressed in islets, and there were also significant differences in the expression levels of various subtypes. The muscarinic receptor M3 and adrenergic receptor α2a were the highest in this study among all of the subtypes according to the results of RT‐PCR and immunohistochemical staining. Immunofluorescence co‐staining was further performed, and the results demonstrated that the muscarinic receptors M3 and M5 were highly expressed in β‐cell, M1 and M3 were costained with α‐cell, and M1 and M4 were localized to δ‐cell (Figure 2C,E,F). However, all 6 adrenergic receptor subtypes of the sympathetic neurotransmitter NE were highly costained with β‐cell, and only α2b‐AR and α2c‐AR were expressed in α‐cell and δ‐cell (Figure 2D,F). Various receptor subtypes were also expressed in different islet cell lines (Figure S1D,E,F), and the physiological concentration (10 μmol/L) of neurotransmitters was determined and used in this study (Figure S2A,B).

### 3.3 MS‐based quantitative proteomic and phosphoproteomic characterization of murine islets treated with ACh/NE

**FIGURE 3:**
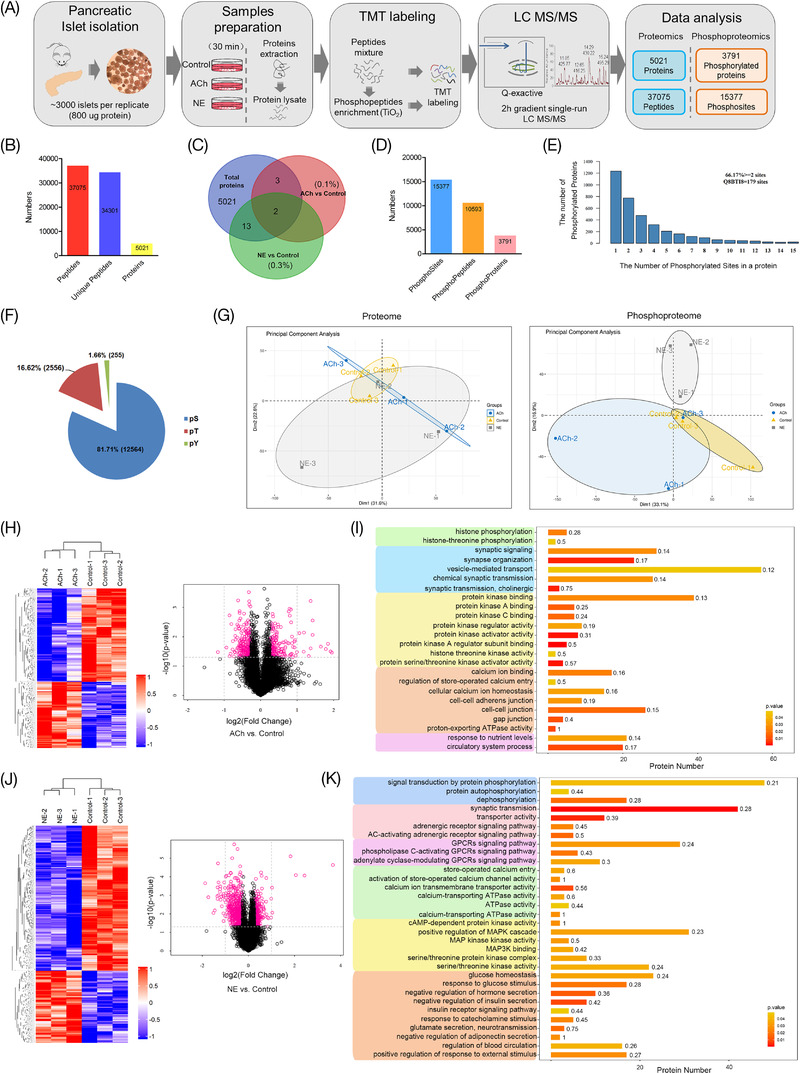
Quantitative proteomics and phosphoproteomic analysis of mouse islets treated with the neurotransmitters acetylcholine (ACh) or norepinephrine (NE) for a short time. (A) Islet sample processing and flowcharts of the quantitative proteomics and phosphoproteomic analyses in this study. (B,C) Numbers of peptides, proteins and significantly different proteins identified by quantitative proteomics in islets treated with ACh or NE. (D) The results of phosphosites, phosphopeptides and phosphoproteins identified by quantitative phosphoproteomic analysis. (E) The number of phosphorylated proteins in the islets after short‐term intervention with ACh and NE. (F) The numbers and proportions of p‐Ser, p‐Thr and p‐Tyr residues in phosphorylated proteins of islets. (G) Principal component analysis (PCA) of proteins and phosphorylated proteins in mouse islets treated with different neurotransmitters by proteomics and phosphoproteomics. (H,J) The results of clustering analysis are presented as heatmaps and volcano plots (log2‐fold change) and ‐log10 (*p*‐value). (I,K) Gene ontology (GO)‐based enrichment analysis revealed the biological processes in which the significantly different phosphoproteins were involved

To elucidate a comprehensive perspective of the molecular mechanisms underlying neurotransmitter‐stimulated islet hormone secretion, deep MS‐based quantitative proteomic and phosphoproteomic profiling was performed in murine pancreatic islets in this study (Figure 3A). As shown in Figure 3B,C, the results of the quantitative proteomic analysis showed that a total of 5021 types of proteins and 37 075 peptides were identified in murine islets. The significantly changed proteins in islets treated with ACh and NE were only 0.1% and 0.3%, respectively. The PCA results also demonstrated that there was no obvious difference in the principal component analysis between groups treated with neurotransmitters (Figure 3G and Figure S3A,B). However, 3791 types of phosphorylated proteins, 10 593 phosphorylated peptides and 15 377 phosphorylation sites were identified in the islets that were treated with neurotransmitters after phosphoproteomic analysis (Figure 3D). In addition, 66.17% of proteins had multiple sites (two or more), and 179 phosphorylation sites were found in protein Q8BTI8 (Figure 3E). Among all of the phosphorylation sites, the proportions of serine, threonine and tyrosine were 81.71%, 16.62% and 1.66%, respectively (Figure 3F). PCA of phosphorylated proteomics also demonstrated that the principal components of islets were significantly different from those of the control group after ACh or NE treatment, indicating that the phosphorylation levels of islet proteins were changed significantly after short‐term stimulation with neurotransmitters (Figure 3G). The cluster analysis results also revealed that 187 significantly hyperphosphorylation proteins and 265 hypo phosphorylation proteins were found in the islets treated with ACh, and 309 hyperphosphorylation proteins and 622 hypo phosphorylation proteins were found in the NE‐treated group (Figure 3H,J). The analysis of GO functional enrichment demonstrated that all of these proteins with significantly changed phosphorylation levels participated in various biological processes, such as synaptic signal transduction, the GPCR signalling pathway, activation of kinase activity and calcium channel opening (Figure S3C,D). Furthermore, the results also revealed that phosphorylated proteins might participate in the response to nutrient levels and the circulatory system in islets after ACh stimulation and positive regulation of the response to external stimuli after NE administration (Figure 3I,K).

### 3.4 Identification of signalling pathways and kinase‐substrate motif enrichment analysis in phosphosites of ACh and NE

**FIGURE 4:**
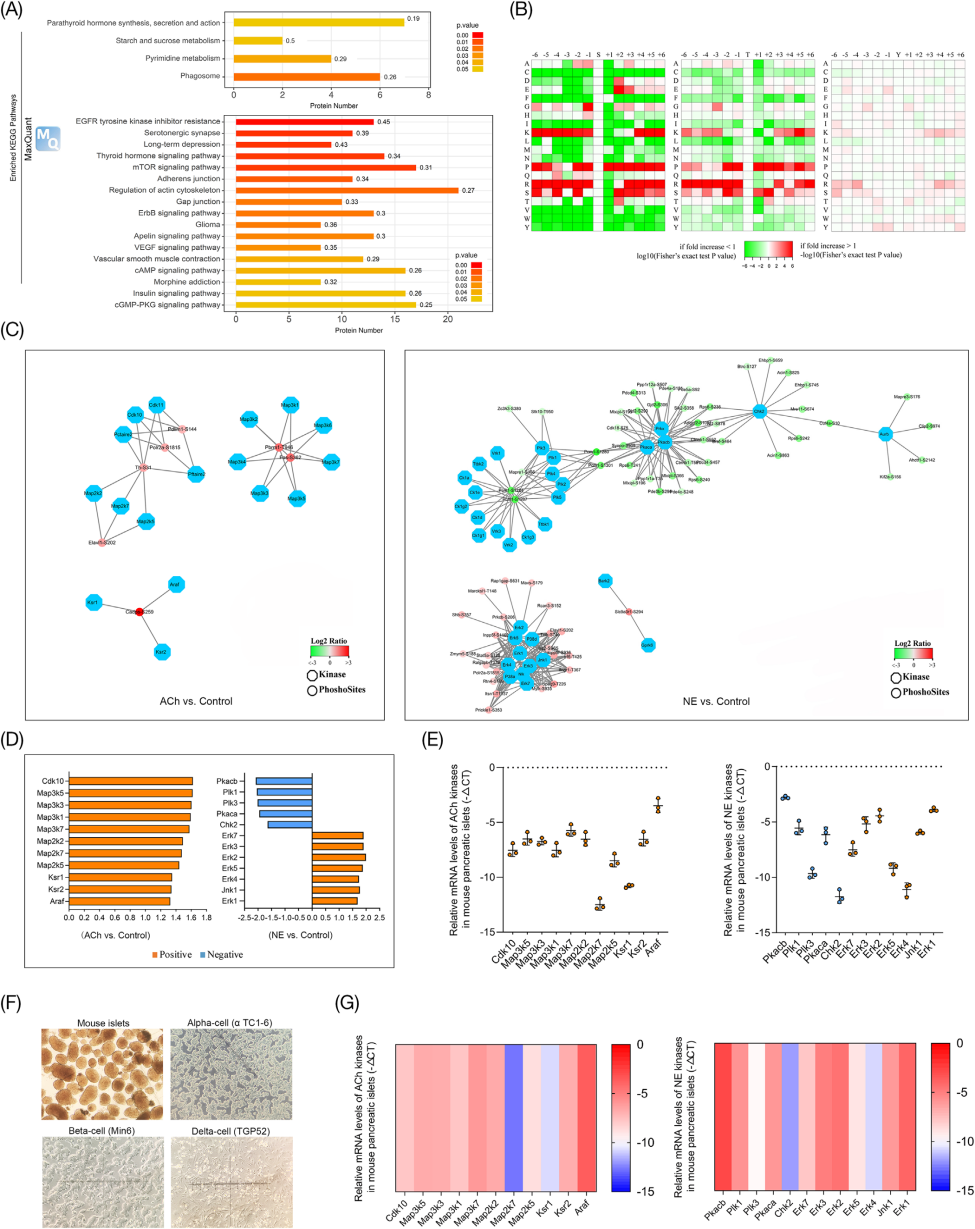
Signaling pathways and key kinases of neurotransmitters acetylcholine (ACh) and norepinephrine (NE) predicted by enriched Kyoto Encyclopedia of Genes and Genomes (KEGG) pathway analysis and motif analysis. (A) Enriched KEGG pathway analysis was performed using the MaxQuant computational platforms for mass spectrometry (MS)‐based phosphoproteomics data in the ACh and NE groups. (B) Motif analysis of phosphosites and phosphoproteins was used to predict the key kinases of the neurotransmitters ACh and NE that regulated the phosphorylation of islet proteins; orange graphs indicate that kinase activity was activated, and blue graphs indicate inhibited kinase activity. (C) The regulatory relationship between kinases and phosphorylated substrate proteins in the ACh and NE groups predicted by motif analysis and presented in the form of the network; red phosphosites indicate the hyperphosphorylation, while green indicates hypo phosphorylation. (D,E) The expression levels of different kinases of ACh and NE predicted by motif analysis were measured by real‐time polymerase chain reaction (RT‐PCR) in mouse islets. (F) Isolated mouse pancreatic islets and three types of pancreatic islet cell lines (alpha TC1‐6, Min6 and TGP52) under an optical microscope (x100 magnification). (G) The heatmaps showing the expression of kinases predicted by motif analysis

In this study, proteins with significantly changed phosphorylation levels were also analyzed using the KEGG database in the MaxQuant and Perseus environments to reveal the signalling pathways of the neurotransmitters ACh and NE. As shown in Figure 4A, the results demonstrated that the phosphorylated proteins in islets were involved in many signalling pathways, such as starch and sucrose metabolism, pyrimidine metabolism and type 2 diabetes mellitus after ACh stimulation (both *p* < 0.05, Figure 4A and Figure S4A). In the NE‐treated islets, potentially involved pathways were more abundant, such as the insulin signalling pathway, vascular smooth muscle contraction, and cAMP and cGMP‐PKG pathways (Figure 4A and Figure S4B). Additionally, kinase‐substrate motif enrichment analysis of phosphosites in murine islets was also performed after stimulation with ACh or NE to investigate the upstream cognate kinases based on iGPS and GSEA methods (log2‐fold change of the expression levels, Figure 4B). In the ACh‐treated group, a total of 17 types of potential upstream regulatory kinases were predicted, and the activity of all of these kinases was increased (Figure S5A). Furthermore, a total of 33 types of kinases were found in the NE group, including 12 types of kinases with increased activity and 21 types with decreased activity (Figure S5B). All of the predicted regulatory relationships between kinases and substrates are also mapped and presented as a global kinase‐substrate network according to the results of motif enrichment analysis (Figure 4C). And all of the kinases were further analyzed using multiple databases (GeneCards, UniProt and PhosphoSitePlus) to screen out the kinases that were closely related to hormone secretion (Figure 4D). Then, IPA analysis was also used to reveal the networks between the predicted kinases in islets after treatment with ACh and NE, and the results showed that the kinases Mapk, Erk1/2 and Pkc played important roles in this study (Figure S4C,D). Furthermore, murine islets and various pancreatic islet cell lines (αTC1‐6, Min6 and TGP52) were used for further study in vitro, and the results of RT‐PCR showed that all of the predicted kinases of ACh and NE were highly expressed in islets and cell lines (Figure 4E–G and Figure S5D). Thus, follow‐up functional experiments were performed in our study.

### 3.5 Specific kinases and substrates of neurotransmitters ACh and NE verified in different islet endocrine cells

**FIGURE 5:**
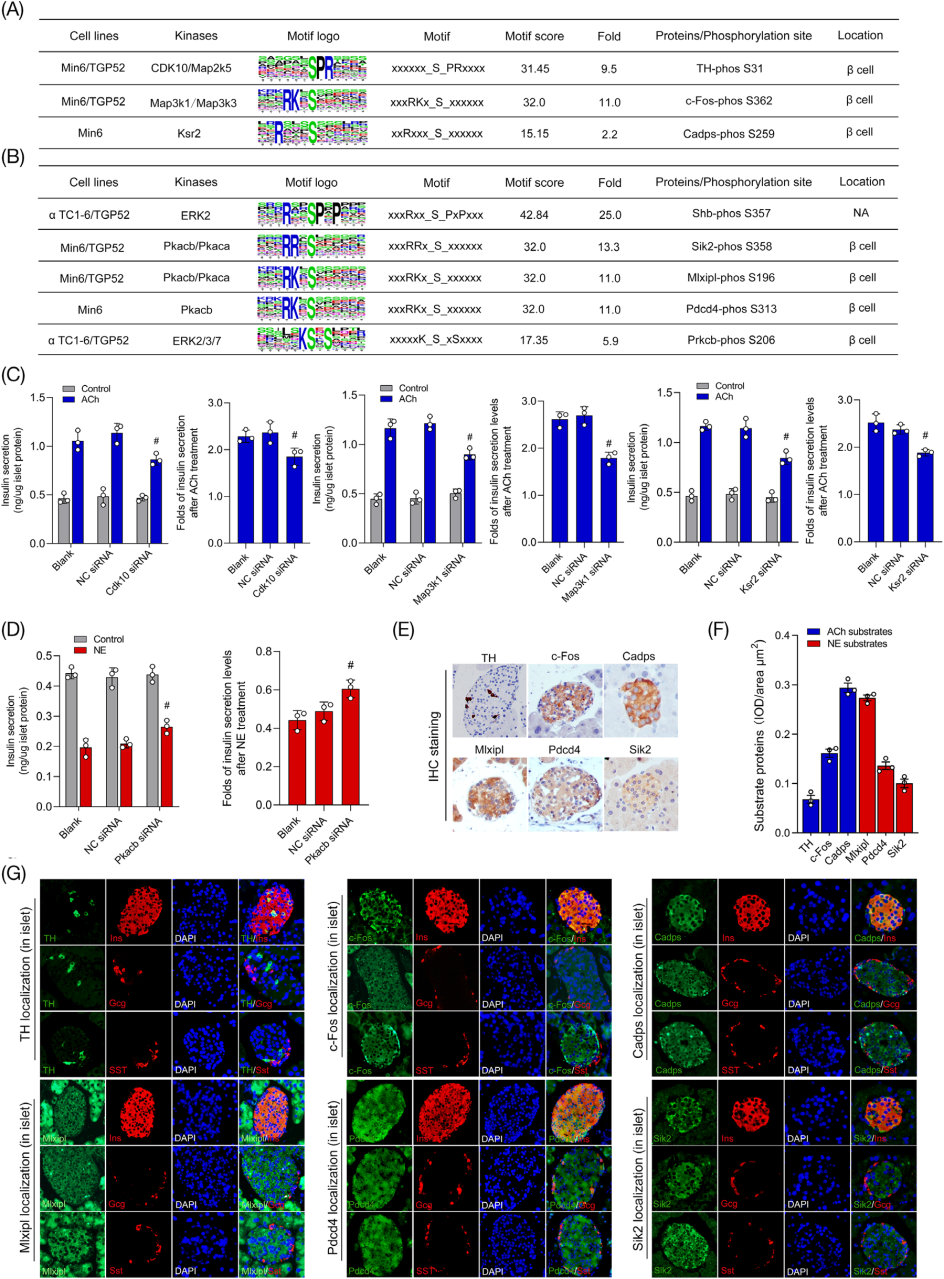
The function and location of key kinases were further verified in murine islets and three different pancreatic cell lines. (A,B) Summary of key phosphorylation kinases, phosphorylated proteins and phosphosites of acetylcholine (ACh) and norepinephrine (NE) according to motif analysis and immunofluorescence co‐staining in different types of islet cells. (C,D) Specific small‐interfering RNAs (siRNAs) were used to interfere with kinase expression in three types of pancreatic islet cell lines to determine the key kinases involved in the regulatory roles of ACh and NE in the secretion of insulin. (E) The expression of key substrate proteins of ACh (TH, c‐Fos and Cadps) and NE (Mlxipl, Pdcd4 and Sik2) measured by immunohistochemical staining in murine islets. (F) Quantitative analysis of substrate proteins of neurotransmitters ACh and NE expressed in the islet. (G) The expression and distribution of substrate proteins in islet endocrine cells were analyzed by colocalization with glucagon (alpha‐cell), insulin (beta‐cell) and somatostatin (delta‐cell) by immunofluorescence multistaining

The potential kinases of the neurotransmitters ACh and NE were predicted by motif enrichment analysis of phosphosites in this study, and the results of real‐time RT‐PCR showed that all of the kinases were highly expressed in the murine islet and three types of islet endocrine cell lines. The results also revealed that the receptors for ACh and NE were expressed in different islet cell lines and that both neurotransmitters could significantly modulate the secretion of various islet hormones. Thus, the specific interferences on kinases by siRNAs were performed in islet cell lines (αTC1‐6, Min6 and TGP52). As shown in Figure 5A–D, the results of our study demonstrated that the secretion levels of islet hormones were evidently decreased after inhibition of the key kinases in different cell lines. The kinases Cdk10, Map3k1 and Ksr2 were found to be involved in insulin secretion in the beta‐cell line (Min6) after ACh stimulation (Figure S6A), and the kinases Map2k5 and Map3k3 were found in delta cells (TGP52, Figure S6b). Additionally, the kinases Erk2/3/7 and Pkaca were considered the key kinases of NE in the αTC1‐6 and TGP52 cell lines (Figure S7A,B), and Pkacb was found in the Min6 cell line (Figure S7C). Then, immunohistochemical staining and immunofluorescence co‐staining were both performed to further verify the expression and localization of the substrate proteins and functional kinases in the murine islet (Figure 5E,G). It is considered a meaningful functional kinase‐substrate relationship only when the substrate protein and its upstream regulatory kinases are detected in the same specific endocrine cell line. The results of this study showed that the substrates of ACh (TH, c‐Fos and Cadps) and NE (Mlxipl, Pdcd4 and Sik2) were highly expressed in islets and were especially costained with beta‐cells (Figure 5F,G). Thus, the Cdk10, Map3k1, Ksr2 and Pkacb found in the Min6 cell line were considered the key regulatory kinases of the neurotransmitters ACh and NE.

### 3.6 Aberrant regulation of neurotransmitters regarding islet hormone secretion in diabetic mice with different islet functions

**FIGURE 6:**
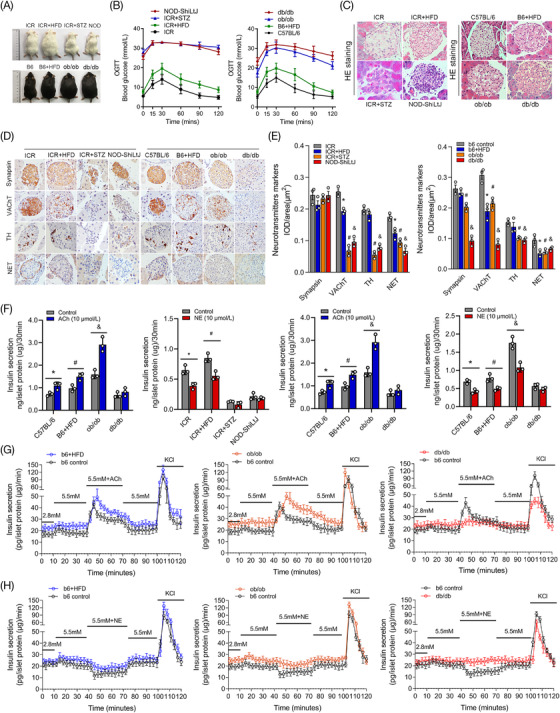
Aberrant regulation of neurotransmitters regarding islet hormone secretion was closely related to islet dysfunction in different diabetic mice. (A,B) Diabetic mouse models with different degrees of islet dysfunction were established and grouped according to islet function in this study. (C) HE (hematoxylin‐eosin staining) staining of pancreatic islets in different diabetic mice was performed to determine the pathological changes. (D,E) The expression of specific markers of the neurotransmitters acetylcholine (ACh) and norepinephrine (NE) measured by immunohistochemical staining and the mean integrated optical density (IOD)/area of the pancreatic islet in different diabetic groups. *p < 0.05: high‐fat‐diet (HFD) group versus the control group, #*p* < 0.05: STZ group or ob/ob group versus the control group, &*p* < 0.05: NOD group or db/db group versus the control group. (F) Insulin secretion levels of murine islets in different diabetic mouse groups were measured under short‐term stimulation with ACh or NE in vitro. *p < 0.05: ACh or NE group versus the control group, #*p* < 0.05: HFD group versus the control group. (G,H) The rapid insulin secretion ability in diabetic mice with various degrees of islet dysfunction was revealed by islet perfusion under stimulation with different neurotransmitters

To further explore whether the effects of the neurotransmitters ACh and NE on islet hormone secretion were abnormal under pathological conditions, various diabetic mice with different islet functions were established in this study (Figure 6A,B). The results of pathological staining also revealed that there were significant differences in the morphological and structural changes of islets in diabetic mice with diverse islet functions (Figure 6C). The structure of the islets in mice with poor islet function was obviously damaged. As shown in Figure 6D,E, the results of immunohistochemical staining for specific markers of ACh and NE also demonstrated that the expression levels of VAChT, TH and NETs were significantly decreased in the islets of diabetic mice, especially in mice with poor islet function. However, synapsin was found to be increased in the islets of type 1 diabetic model mice (STZ and NOD groups). Islet hormone secretion was also measured in isolated diabetic mouse islets stimulated with the neurotransmitters ACh or NE in vitro, and increased insulin secretion was found in the control group, HFD group and ob/ob group after ACh treatment, while this outcome was not evident in the STZ group, NOD group or db/db group. The inhibitory effect of NE on insulin secretion was also found to be significantly decreased in the islets of mice with poor islet function (STZ, NOD and db/db mice, Figure 6F and Figure S8A,B). Furthermore, the results of islet perfusion demonstrated that the levels of insulin secretion were evidently changed after neurotransmitter ACh or NE stimulation in the HFD and ob/ob groups, and this change was not significant in the db/db mice (Figure 6G,H). However, although the pancreatic islets of HFD and ob/ob mice could respond significantly to ACh or NE treatment, there was also an obvious delay in the peak insulin secretion compared with the control group. The plateau phase of hormone secretion during neurotransmitter maintenance also disappeared in diabetic mice with poor islet function.

### 3.7 Functional receptors, kinases and substrates of ACh and NE expressed in the islets of diabetic mice

**FIGURE 7:**
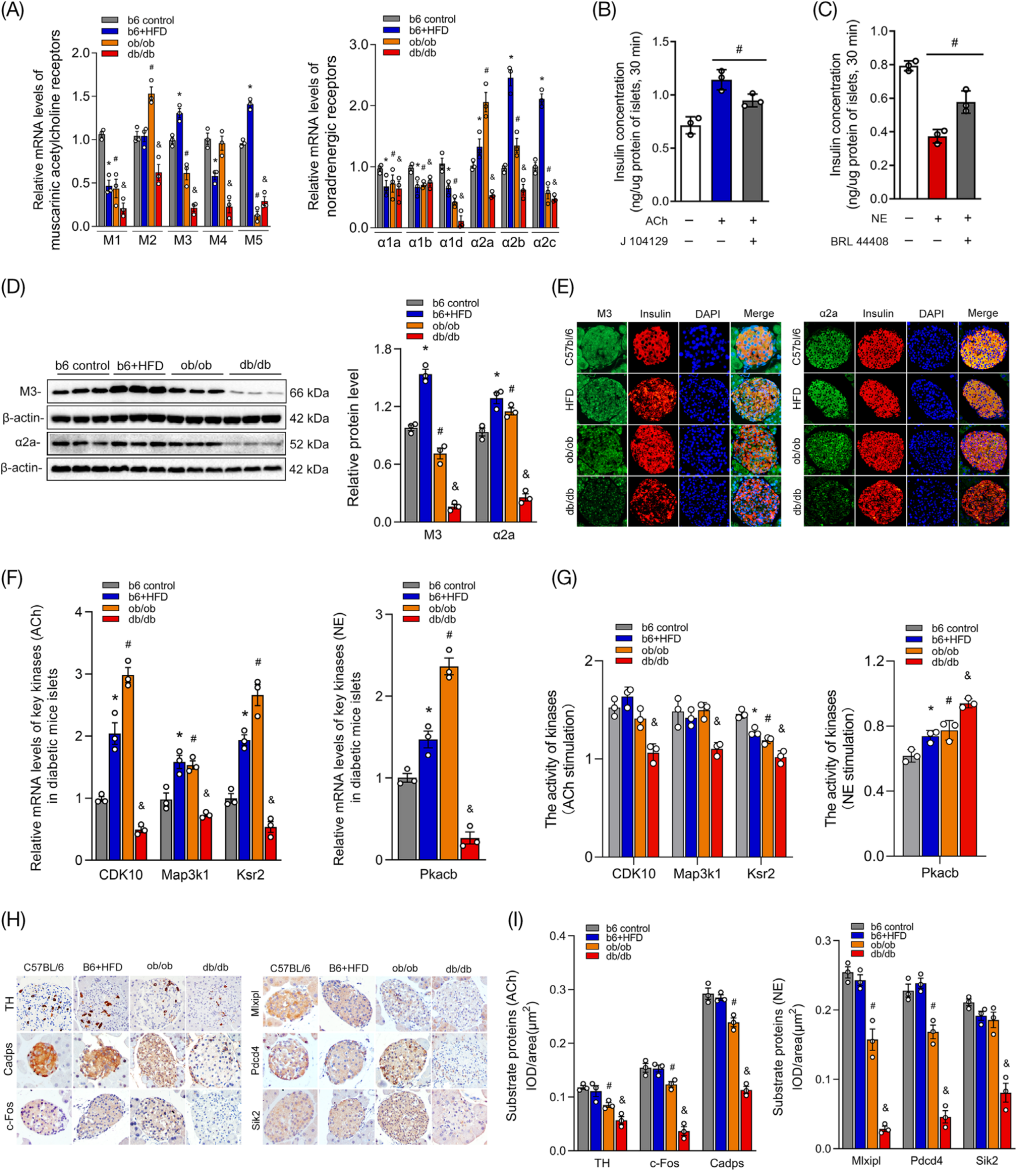
The expression levels of receptors, kinases and substrates of neurotransmitters in the islets of various diabetic mice. (A) Various receptor subtypes of the neurotransmitters acetylcholine (ACh) and norepinephrine (NE) expressed in different diabetic mice were measured by real‐time polymerase chain reaction (RT‐PCR). (B,C) The specific antagonists J104129 and BRL44408 were used to evaluate the roles of receptors M3 and α2a in insulin secretion after stimulation with ACh or NE. (D) The expression levels of M3 and α2a were measured by western blot analysis and immunofluorescence co‐staining with insulin in the islets of different diabetic mice. (E,F) Relative mRNA levels of key kinases of ACh (Cdk10, Map3k1 and Ksr‐2) and NE (Pkacb) in murine islets. (G) The activities of kinases in the islets of diabetic mice after stimulation with ACh and NE. (H) Immunohistochemical staining of substrate proteins of ACh (TH, Cadps and c‐Fos) and NE (Mlxipl, Pdcd4 and Sik2) in the islets of different diabetic mice. (I) Quantitative analysis of substrate proteins was measured by the mean integrated optical density (IOD)/area in diabetic mouse islets. *p < 0.05: high‐fat‐diet (HFD) group versus the control group, #*p* < 0.05: ob/ob group versus the control group, &*p* < 0.05: db/db group versus the control group

Previous results in this study indicated that the neurotransmitters ACh and NE regulate islet hormone secretion mainly through binding with multiple receptors. Thus, real‐time RT‐PCR was performed to evaluate the expression of various receptor subtypes in diabetic mice, and we found that all of the receptors of ACh and NE were markedly decreased in the islets of diabetic mice with poor islet function, such as STZ mice, NOD mice and db/db mice. However, several receptor subtypes were also found to be increased in HFD mice or ob/ob mice (Figure 7A). The results also revealed that the expression of the receptors M3 and α2a was significantly higher than other subtypes in islet, and the regulatory effects of ACh and NE on insulin secretion were also significantly decreased after using specific antagonists of M3 (J104129, Figure 7B) and α2a (BRL44408, Figure 7C). Then, the results of western blotting analysis and immunofluorescence co‐staining demonstrated that the expression of M3 and α2a was evidently decreased in diabetic mice with poor islet function (db/db mice, Figure 7D,E). Additionally, key kinases of the neurotransmitters ACh and NE were detected in this study, and the results showed that the expression levels of these key kinases were increased in the islets of HFD mice and ob/ob mice but significantly decreased in those of db/db mice (Figure 7F). The activity of kinase Ksr2 activity was decreased in diabetic mice after ACh stimulation (both *p* < 0.05), but the kinase Pkacb was abnormally activated after NE treatment (Figure 7G). In addition, the expression of substrate proteins of ACh and NE were also measured by immunohistochemical staining, and the results showed that substrate proteins were significantly decreased in the islets of ob/ob and db/db mice, but this outcome was not evident in the HFD mice (Figure 7H,I).

### 3.8 Abnormal rhythm of neurotransmitter‐mediated hormone secretion was ameliorated by combined intervention with receptors and key kinases

**FIGURE 8:**
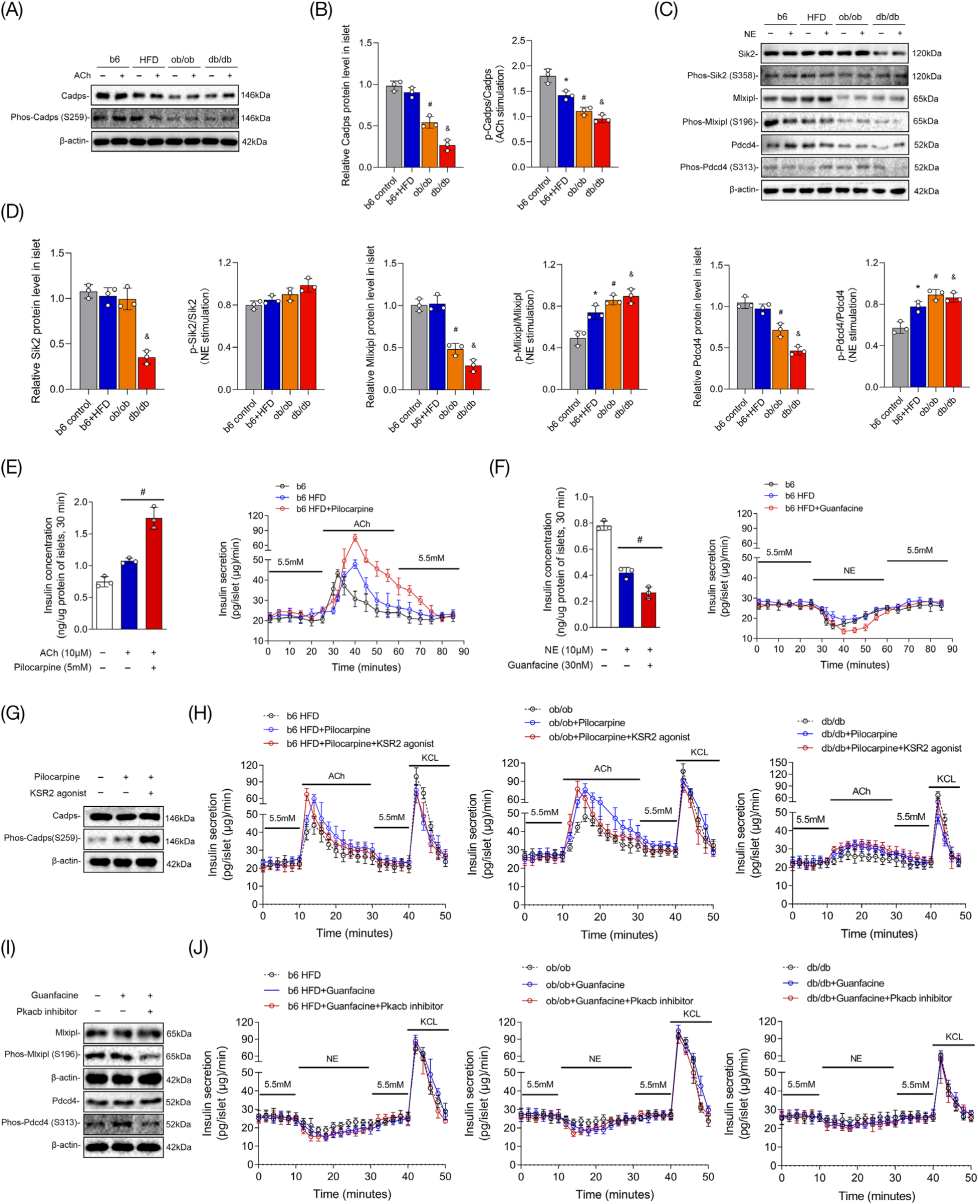
Combined interventions on receptors and key kinases in different diabetic mice. (A,B) The expression and phosphorylation of substrate Cadps measured by western blot analysis after acetylcholine (ACh) stimulation in different diabetic mice; (C,D) The expression levels and phosphorylation of Sik2, Mlxipl and Pdcd4 after norepinephrine (NE) stimulation; (E) The level of insulin secretion was measured in the islets of high‐fat‐diet (HFD) mice treated with cholinergic receptor M3 agonist and ACh; (F) The insulin secretion in HFD mice after treatments with α2a agonist and NE; (G) The phosphorylation of the substrate protein Cadps after combined interventions on receptor M3 and Ksr2 activity; (H) The insulin secretion of islets in different diabetic mice after administration of pilocarpine and Ksr2 agonist revealed by islet perfusion; (I) The phosphorylation of the substrate proteins Mlxipl and Pdcd4 after interventions with α2a agonist and Pkacb inhibitor; (J) Insulin secretion in different diabetic mice after treatments with guanfacine and Pkacb inhibitor

Islet protein phosphorylation mediated by kinases plays an important role in rapid hormone secretion after neurotransmitter stimulation. Pathological staining also revealed that the expression of substrates and kinases was closely associated with the degree of islet dysfunction in diabetes mellitus. As shown in Figure 8A–D, the results of western blotting demonstrated that Cadps, Sik2, Mlxipl and Pdcd4 were significantly decreased in the islets of diabetic mice with poor islet function (ob/ob mice and db/db mice). Although substrate expression was not evidently decreased in the islets of HFD mice, protein phosphorylation was impaired after neurotransmitter stimulation. Then, receptor agonists of M3 (pilocarpine, Figure 8E) and α2a (guanfacine, Figure 8F) were used in this study, and the results showed the effects of neurotransmitters on insulin secretion were enhanced significantly, while the delayed peak of the valley still existed in insulin secretion. As shown in Figure 8G–J, the results showed that the phosphorylation of substrate proteins was not evidently improved after using the receptor agonists of M3R and α2a. However, combined interventions with receptor and kinase activities by agonists or antagonists had significant effects on substrate protein phosphorylation (Figure 8G,I). Furthermore, islet perfusion also demonstrated that the abnormal rhythm in rapid insulin secretion after neurotransmitter stimulation was also improved after the combined administration of receptors and kinases in HFD mice and ob/ob mice. However, this phenomenon was not evident in the db/db mice (Figure 8H,J).

## 4 DISCUSSION

The pancreatic islet is highly innervated by abundant autonomic nerves, playing a critical role in systemic energy metabolism and blood glucose homeostasis.^31^ Neurotransmitters are important mediators of islet function during the modulation of the brain, and this process mainly depends on various receptors expressed on endocrine cells.^6^ In this study, rapid regulatory effects of antagonistic neurotransmitters NE and ACh on islet hormone secretion were measured, and the downstream molecules and mechanisms were also explored. The results demonstrated that various receptor subtypes of ACh were expressed in alpha‐cell, beta‐cell and delta‐cell of islets, while the receptors of NE were mainly expressed in beta‐cell. Islet perfusion also revealed that transmitters ACh and NE could rapidly modulate the secretion of different islet hormones, and a significant “platform phase” in the secretion curve was also observed after neurotransmitter administration. Quantitative proteomics and phosphoproteomics were innovatively performed on mouse islets after stimulation with ACh and NE, and the results demonstrated that there was no significant difference in protein expression after neurotransmitter treatments, while the levels of phosphorylation were changed significantly. These proteins are involved in many biological and pathological processes, such as synaptic signal transduction, calcium channel opening and initiation of diabetes mellitus. The functional kinases and substrate proteins of ACh and NE in islets were predicted using motif analysis in this study, and the expression levels of the kinases and substrates were found to be significantly decreased in diabetic mice with poor islet functions. Furthermore, aberrant neural regulation of islet hormone secretion in diabetic mice with early islet dysfunction could be improved by combined administrations of specific receptors and key kinases. This finding could provide a promising route to target islet nerves to ameliorate the islet function of diabetic patients through potential pharmacological treatments in the future.

The autonomic nerves in the islet mainly include the sympathetic nerve from the visceral nerve and the parasympathetic branch from the vagus nerve.^32^ The nerves ending in the pancreas form a dense neural network cluster in the islet through the branches of the intercellular space to regulate the secretion of different islet hormones in response to various physiological cues. The neurotransmitters in the islet mainly bind with different types of receptor subtypes, and all of these receptors are GPCRs. Phosphorylation, as an important posttranslational modification of proteins, plays a critical role in the downstream signal transduction of GPCRs. The phosphate groups provided by ATP are transferred to the amino acid residues of substrate proteins (mainly serine, threonine and tyrosine) under the catalysis of various PKs. The reversible process of phosphorylation is closely related to the activation of many kinases and substrate proteins.^33^ In our study, islet hormone secretion and protein phosphorylation were both significantly changed after a short period of stimulation with neurotransmitters, while the protein expression was almost unchanged. This finding indicates that the regulation of islet hormone secretion mediated by neurotransmitters could mainly depend on posttranslational modification to activate various kinases and substrate proteins, rather than altering the expression levels of islet proteins. Protein phosphorylation and dephosphorylation in islets are closely related to the activation of secretion‐related proteins and the rapid release of hormone vesicles under stimulation by neurotransmitters.

In this study, high‐resolution MS‐based proteomics and phosphoproteomics were applied to describe the phosphorylation events quantitatively occurring in islets stimulated with the antagonistic neurotransmitters ACh and NE. A depth of > 5000 proteins in islets was characterized by comparison with the murine proteomic profiles. Then, we also quantified changes in the phosphorylation status of > 15 000 phosphosites upon short‐term stimulation with neurotransmitters. Through the classified data analysis, a variety of physiological and pathological processes were revealed. Apart from the activation of various phosphorylated kinases and GPCR signalling pathways, it is interesting that an enhanced response to nutrient levels and positive circulatory system processes was found in the islets after stimulation with ACh. The parasympathetic neurotransmitter ACh has been reported to increase the response of islet cells, especially β cells, to glucose stimulation, but the mechanism remains unclear.^34^ The secretion of insulin is similar to that of pancreatic juice, and there is also a “cephalic phase” during the process of food intake, which is considered to be mainly innervated by the parasympathetic nerves, and insulin begins to be secreted within 2–3 min before dining.^35^ This phenomenon was also revealed by fake food intake in mice and appetite or olfactory stimulation in the healthy population.^36^, ^37^ In our study, Ksr2 and Cadps were considered the key kinases and substrate proteins of ACh in insulin secretion. Cadps is a calcium‐dependent secretion activator that is also a highly conserved secretion‐promoting protein that acts as a calcium ion channel sensor and regulates the secretion of calcium‐dependent vesicles by sensing the intracellular Ca^2+^ influx process.^38^ It plays an important role in the formation and stability maintenance of secretory vesicles that are directly involved in the process of vesicle secretion and exocytosis.^39^ Thus, kinase Ksr2 activation and Cadps phosphorylation in islets might be involved in the modulation of the cephalic phase of insulin secretion. Additionally, the phosphorylated proteins after NE stimulation participated in various biological processes, including negative regulation of insulin secretion and positive regulation of response to external stimuli. All of these findings suggest that neurotransmitters in islets could play a pre‐regulatory role in islets, which could in turn cause the islets to prepare in advance and better respond to upcoming energy intake or external stimuli.

Furthermore, emerging evidence has also demonstrated that aberrant innervation in the pancreatic islet is closely related to the initiation and development of the metabolic syndrome and diabetes mellitus.^40^ Studies have revealed that the axons of the autonomic nerve in the pancreas of diabetic patients are intact.^4^ Abnormal innervation of the sympathetic nerve and parasympathetic nerve in islets also mediates different phenotypes of islet dysfunction.^41^ Compared with nondiabetic individuals, the sympathetic nerves lose almost 90% in the islet of type 1 diabetic patients. An impaired inhibitory effect of sympathetic nerves on insulin secretion was also found in patients with long‐term type 1 diabetes mellitus.^22^ The decreased distribution of sympathetic nerves might directly affect islet hormone secretion in different physiological cues, and the occurrence of frequent hypoglycemia will also further aggravate autonomic nerve injury in diabetic patients.^42^ It has also been reported that impaired innervation has been found in patients with type 2 diabetes mellitus.^43^ The responses of beta‐cell and alpha‐cell to fluctuations in blood glucose levels were found to be significantly reduced when the sympathetic and parasympathetic nerves were blocked in the pancreas.^44^ In addition, abnormal regulation of the parasympathetic nerve in the islet is also closely related to the occurrence of many types of metabolic syndromes.^5^, ^8^

However, most of the current studies have mainly focused on receptor intervention by neurotransmitters, and few studies have observed the administration of downstream signalling pathways. Thus, in our study, various receptor subtypes, kinases and substrate proteins of the antagonistic neurotransmitters ACh and NE were identified in murine islets and further investigated in different islet endocrine cells and diabetic mice. The receptor‐kinase‐substrate relationships in the neurotransmitters ACh and NE were also revealed and elaborated by functional experiments and colocalization staining. After verification in different diabetic mice with islet dysfunction, Ksr2 and Pkacb were considered the key kinases of the neurotransmitters ACh and NE in the regulation of insulin secretion, respectively. The critical substrate proteins Cadps and Mlxipl were also highly expressed in the beta‐cell of islets. Furthermore, we also found that decreased expression of receptors and impaired phosphorylation of specific substrate proteins are closely related to the aberrant secretion of insulin in diabetic mice. Although the decline in islet function was not significant in HFD‐induced diabetic mice, impaired regulation of neurotransmitters was observed. The innervation of ACh and NE almost disappeared in diabetic patients with poor islet function, which might be caused by the significant decreases in receptors, kinases and substrates in the islet.

There are also some technical limitations of this study. The neural networks and the association with different endocrine cells in islets can only be examined locally rather than substantial understanding at the whole organ level in vivo. The structure of nerve signal transduction in the pancreas and islet is not intact after islet isolation. Therefore, we are further exploring the regulatory effects of ACh or NE in the whole pancreas using perfusion in vivo, but how to prevent the degradation of these neurotransmitters remains an important issue. In recent years, confocal fluorescence microscopy and 3D panoramic histology technology have been used to scan and reveal the original neural network distribution in the pancreas and islets in diabetic mice.^45^ This imaging technology using an integrated neural network will provide the basis and a promising method for further study of neural signal communication and regulation in pancreatic islets.

**FIGURE 9:**
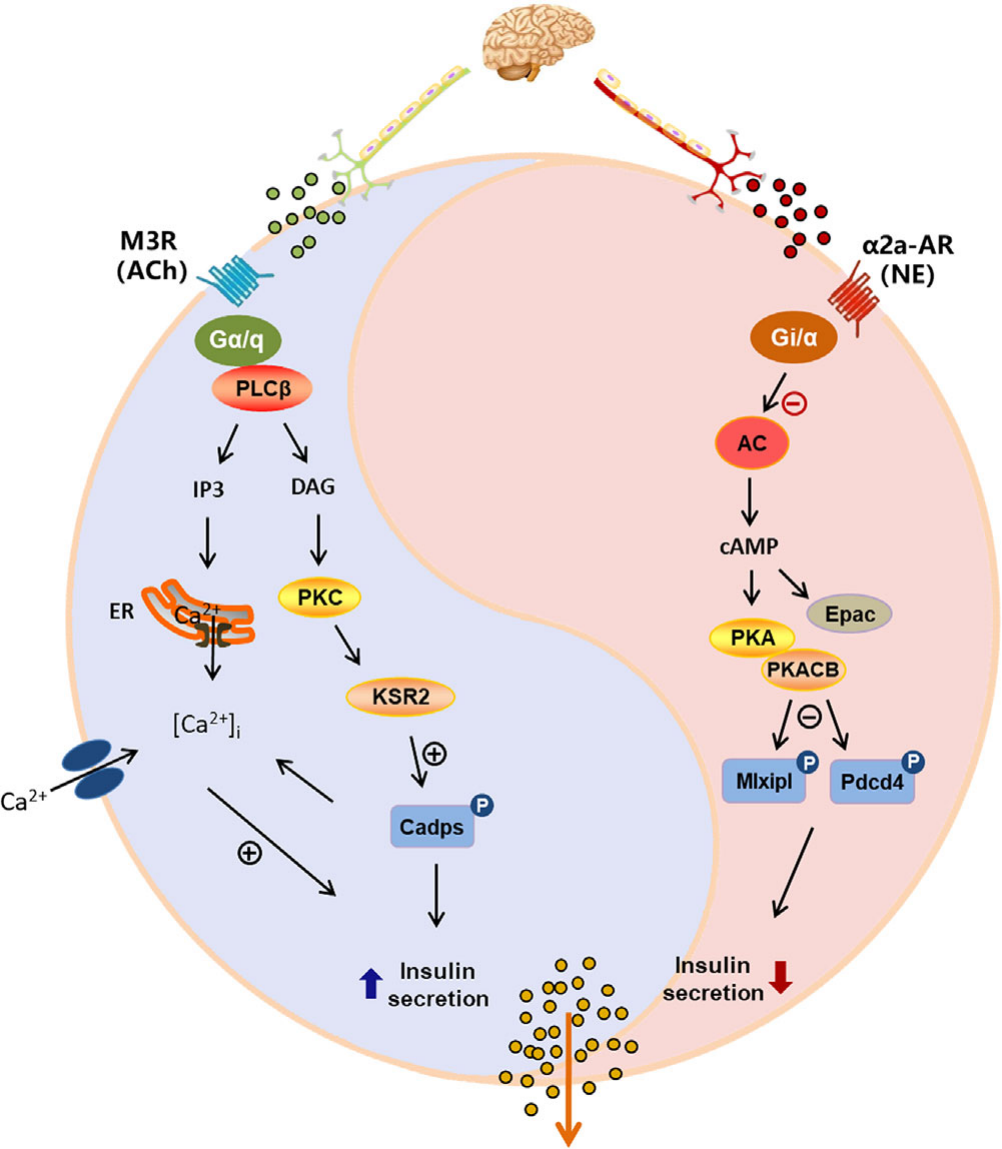
The sympathetic and parasympathetic neurotransmitters norepinephrine/acetylcholine (NE/ACh) coregulate islet hormones secretion through key receptors and kinases in pancreatic islet cell

In conclusion, islet dysfunction is one of the critical pathological mechanisms of type 2 diabetes mellitus, which is mainly manifested by changes in the quality, quantity and rhythm of islet hormone secretion. The abundant autonomic nerves in pancreatic islets play important roles in the regulation of various islet hormone secretion through different types of neurotransmitters. Aberrant regulation of nerves in islet is closely associated with the imbalance of postprandial blood glucose homeostasis. As shown in Figure 9, the antagonistic neurotransmitters ACh and NE in the sympathetic and parasympathetic nervous systems have diverse receptor subtypes in different islet endocrine cells, and they exert rapid regulatory effects on hormone secretion by activating key kinase and substrate protein phosphorylation in islets. Furthermore, impaired innervation in islets is closely associated with islet dysfunction, and the modulation of neurotransmitters regarding islet hormone secretion is significantly weakened or disappears in diabetic mice with poor islet function. Although the decline in islet function was not significant in the prediabetes stage, abnormal innervation already existed in pancreatic islets. Additionally, aberrant neural regulation of hormone secretion in diabetic mice with early islet dysfunction could be improved by the combined administration of specific receptors and key kinases. Thus, in light of the important role of neural signals in the modulation of islet function, neural signals could be considered a promising strategy to target islets for potential pharmacological intervention in diabetes treatment.

## CONFLICT OF INTEREST

The authors declare no conflict of interest.

## Supporting information

Supporting Information

Supporting Information

Supporting Information

Supporting Information

Supporting Information

Supporting Information

Supporting Information

Supporting Information

Supporting Information

Supporting Information

Supporting Information
