# Supplementary figures and images for: Phosphoproteome reveals molecular mechanisms of aberrant rhythm in neurotransmitter‐mediated islet hormone secretion in diabetic mice
Source: Clin Transl Med. 2022 Jun 27;12(6):e890. doi: 10.1002/ctm2.890 (PMC9235066; doi:10.1002/ctm2.890)

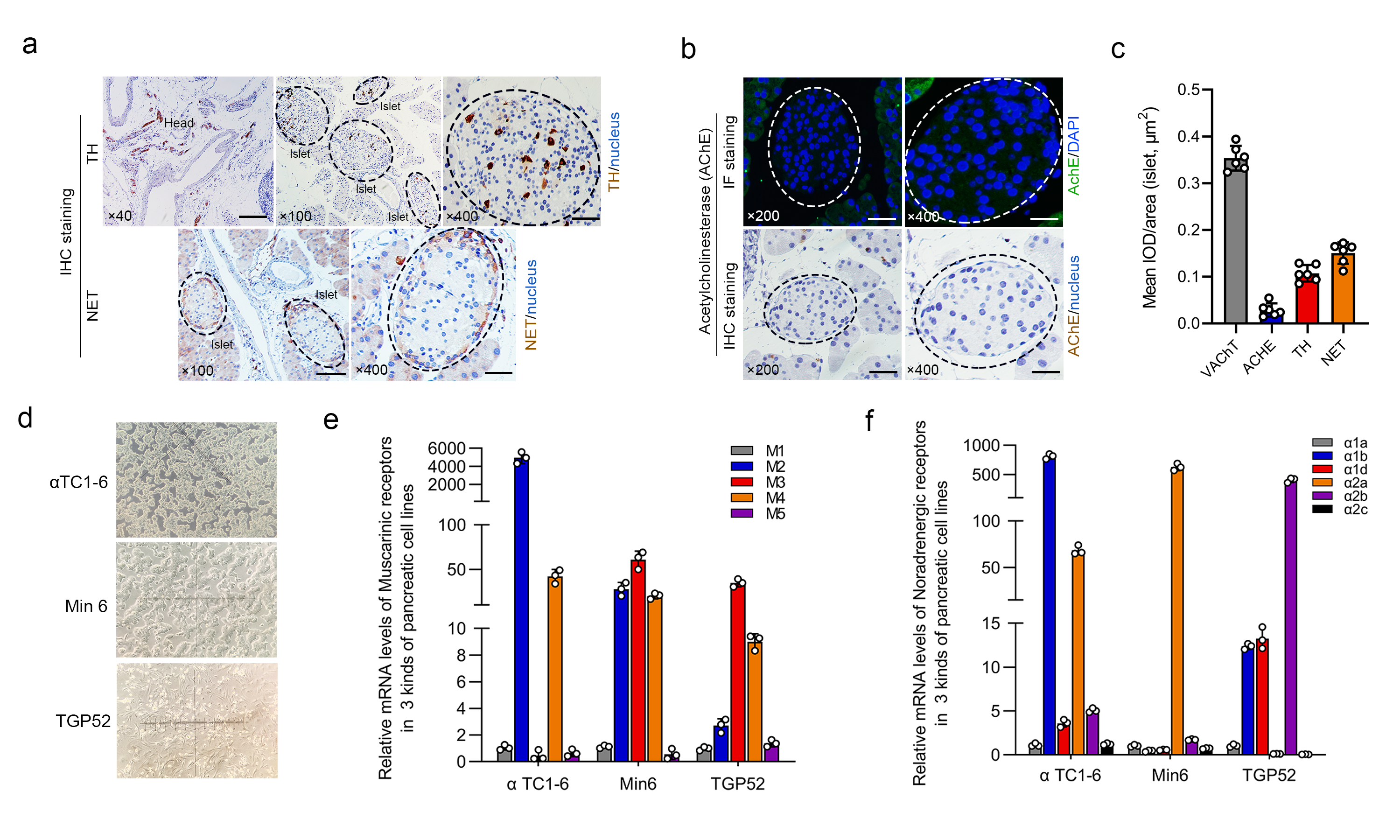

Supplement: Supplementary file 1 — Supporting Information [file CTM2-12-e890-s006.tif]

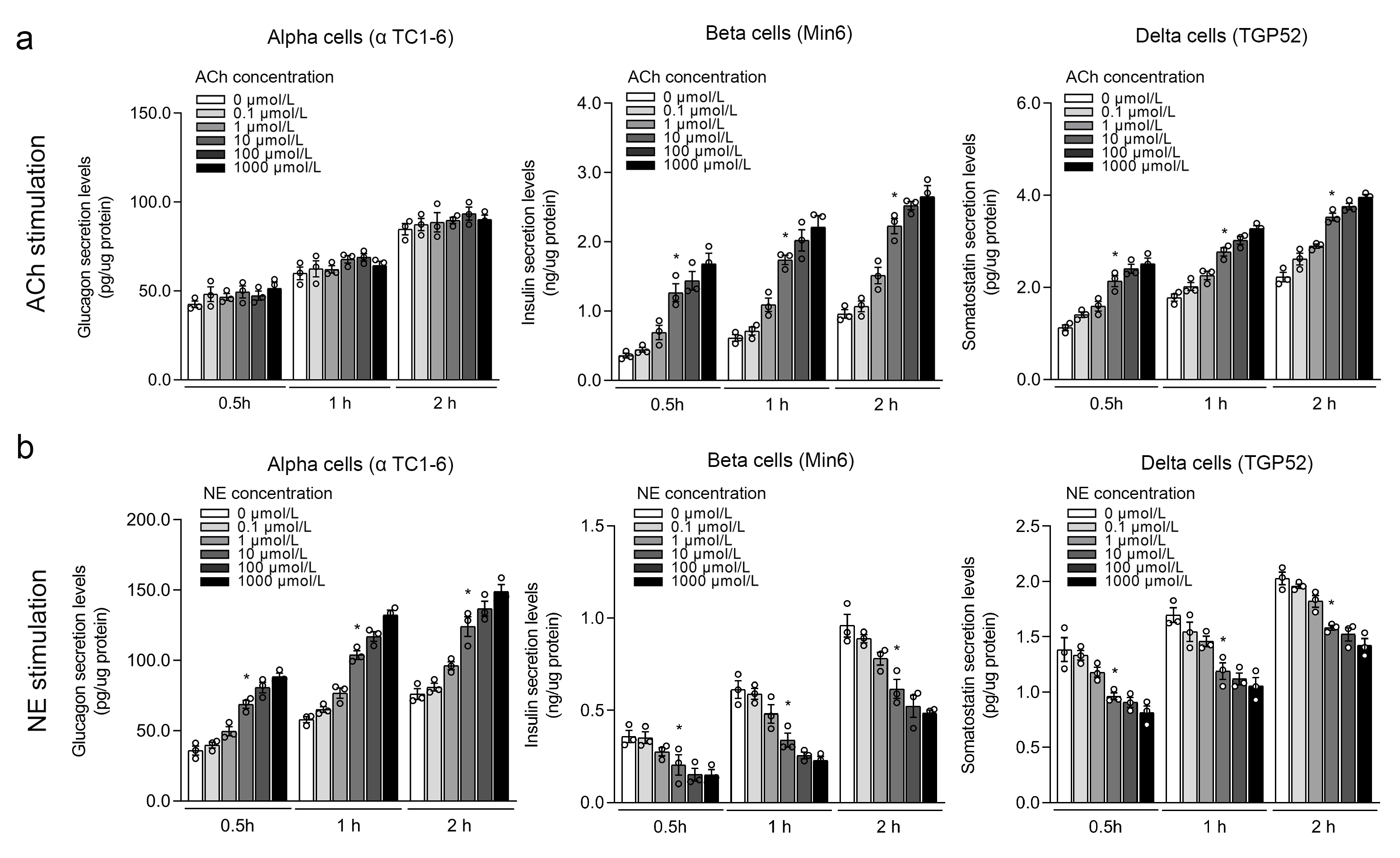

Supplement: Supplementary file 2 — Supporting Information [file CTM2-12-e890-s004.tif]

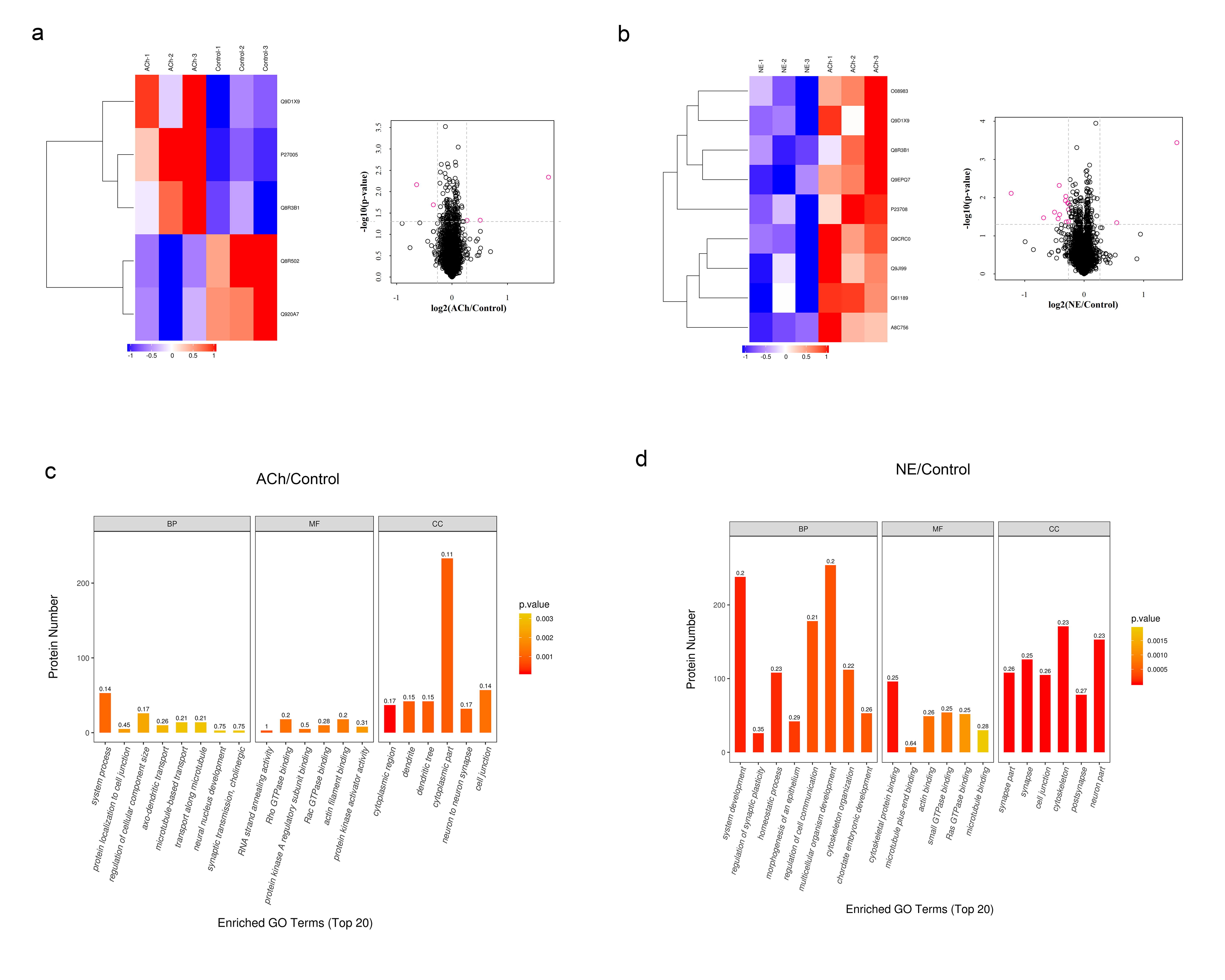

Supplement: Supplementary file 3 — Supporting Information [file CTM2-12-e890-s003.tif]

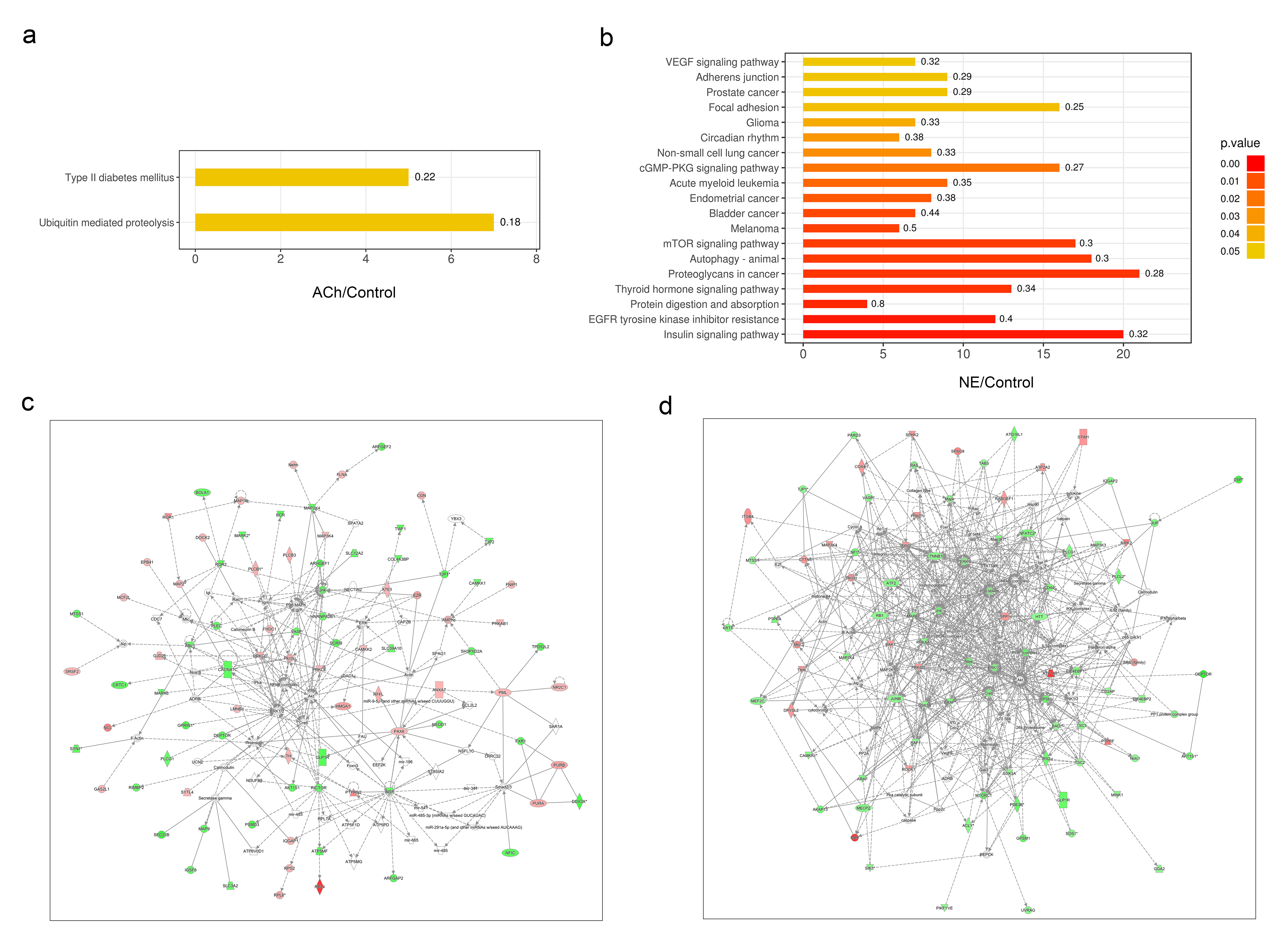

Supplement: Supplementary file 4 — Supporting Information [file CTM2-12-e890-s008.tif]

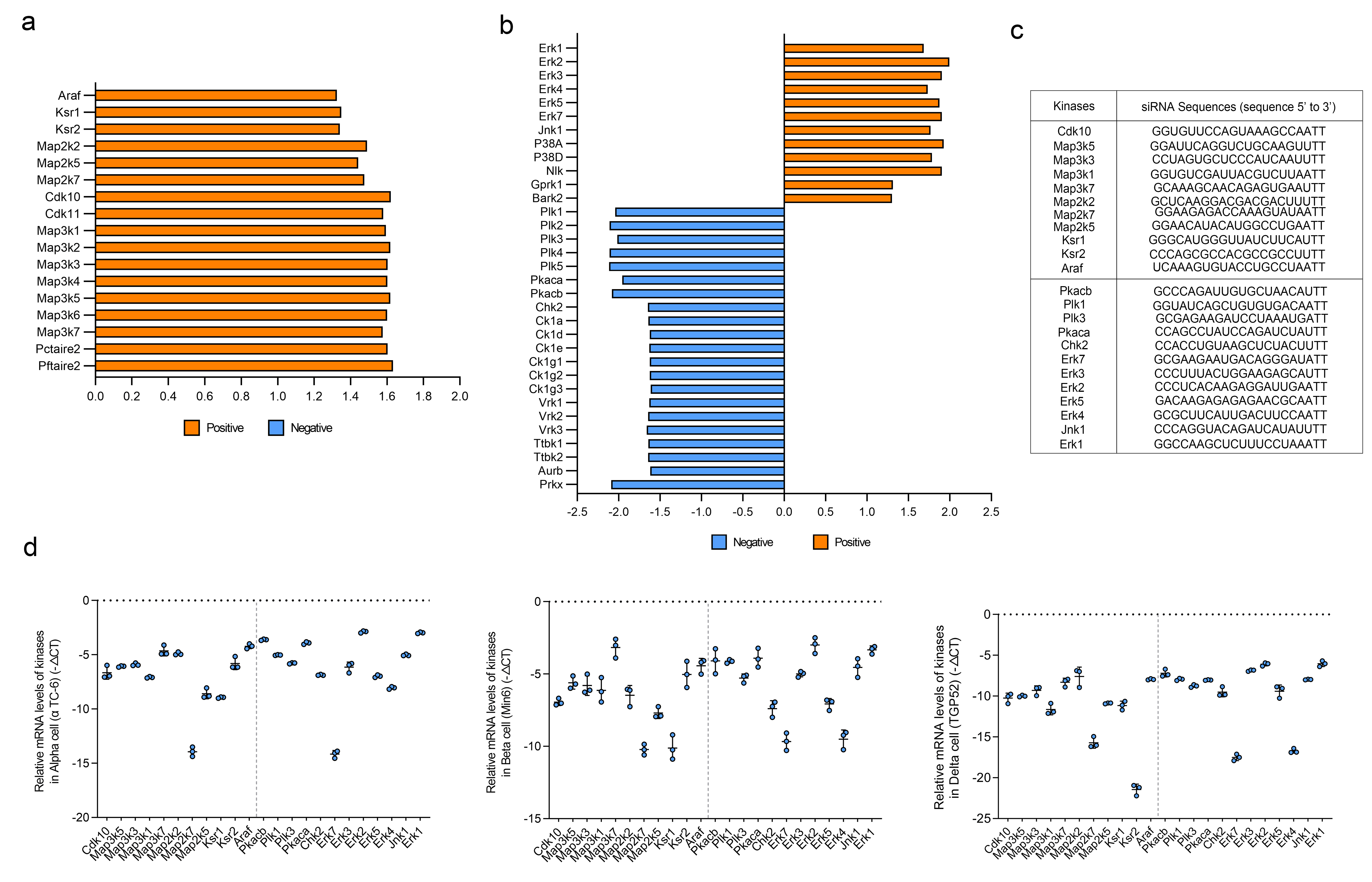

Supplement: Supplementary file 5 — Supporting Information [file CTM2-12-e890-s010.tif]

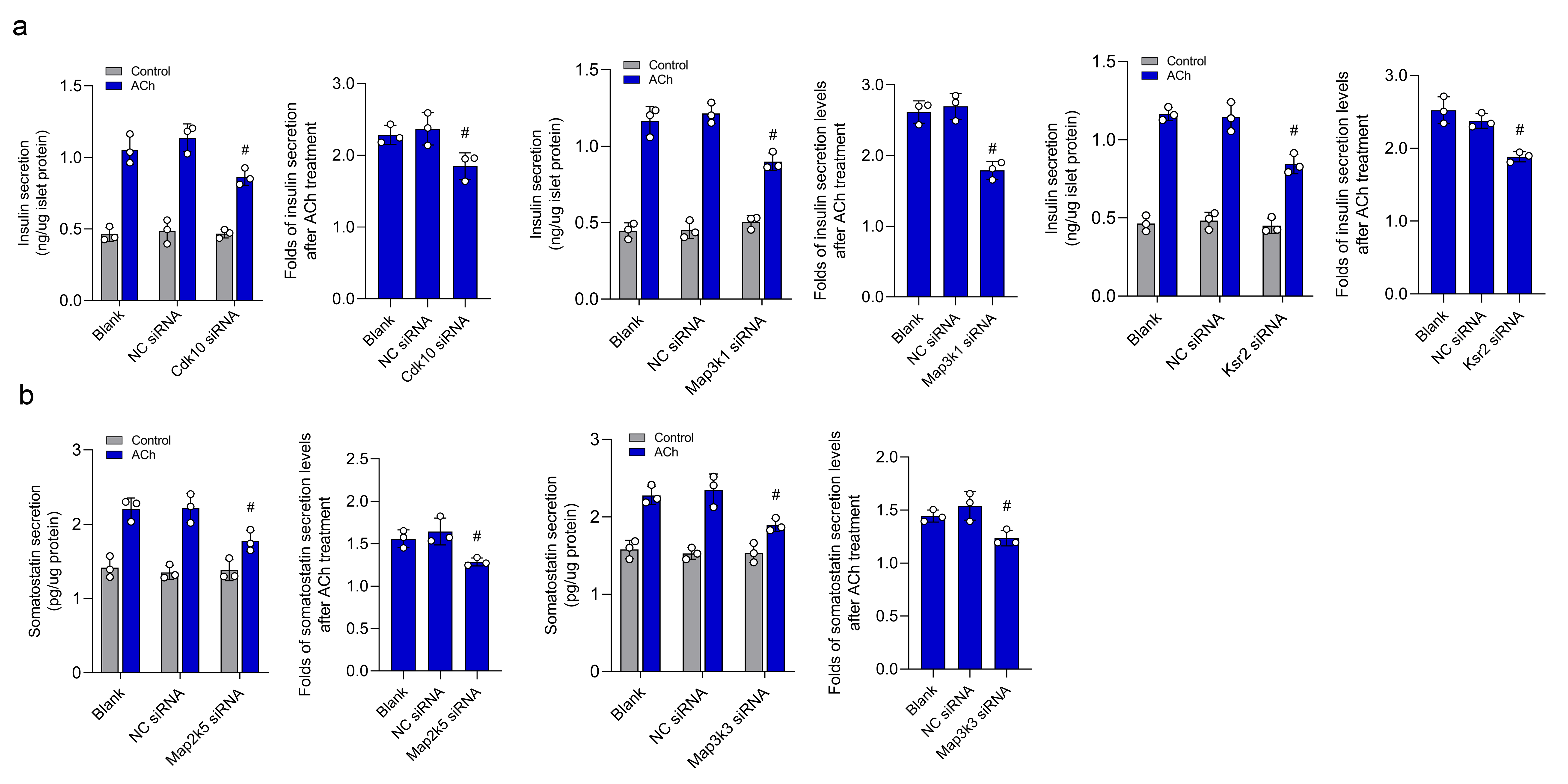

Supplement: Supplementary file 6 — Supporting Information [file CTM2-12-e890-s011.tif]

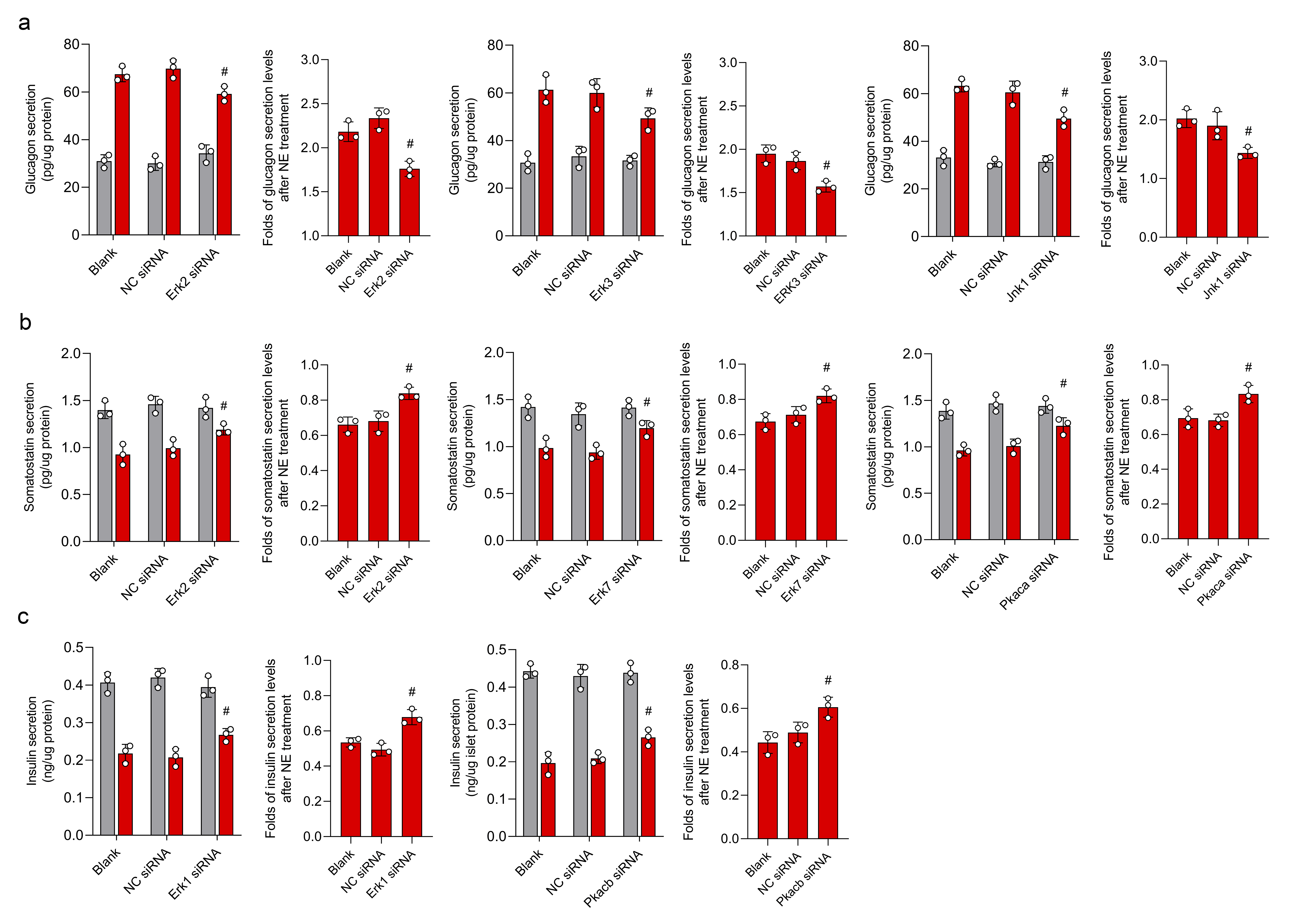

Supplement: Supplementary file 7 — Supporting Information [file CTM2-12-e890-s007.tif]

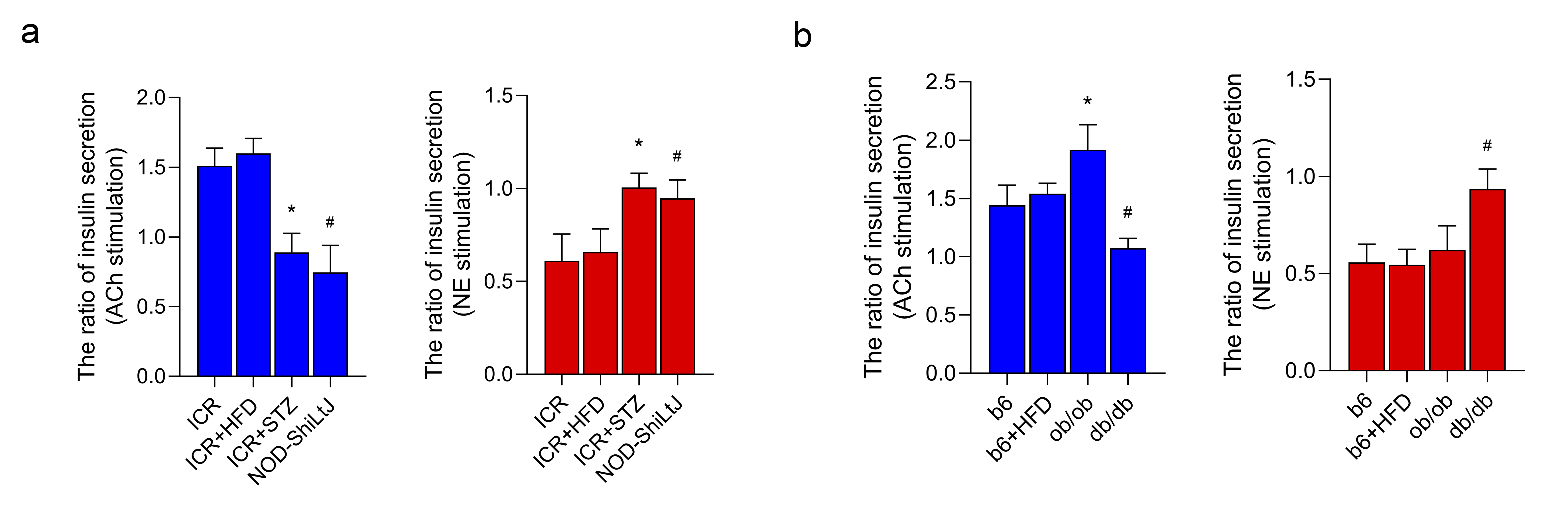

Supplement: Supplementary file 8 — Supporting Information [file CTM2-12-e890-s009.tif]
